# Assessing polygenic risk score models for applications in populations with under-represented genomics data: an example of Vietnam

**DOI:** 10.1093/bib/bbac459

**Published:** 2022-11-02

**Authors:** Duy Pham, Buu Truong, Khai Tran, Guiyan Ni, Dat Nguyen, Trang T H Tran, Mai H Tran, Duong Nguyen Thuy, Nam S Vo, Quan Nguyen

**Affiliations:** Institute for Molecular Bioscience, The University of Queensland, Carmody Rd, 4072, Queensland, Australia; UniSA STEM, University of South Australia, Mawson Lakes, 5095, South Australia, Australia; Center for Biomedical Informatics, Vingroup Big Data Institute, 458 Minh Khai , 10000, Hanoi, Vietnam; Institute for Molecular Bioscience, The University of Queensland, Carmody Rd, 4072, Queensland, Australia; Center for Biomedical Informatics, Vingroup Big Data Institute, 458 Minh Khai , 10000, Hanoi, Vietnam; Center for Biomedical Informatics, Vingroup Big Data Institute, 458 Minh Khai , 10000, Hanoi, Vietnam; Institute for Molecular Bioscience, The University of Queensland, Carmody Rd, 4072, Queensland, Australia

**Keywords:** PRS, GWAS, trans-ethnic, across-population

## Abstract

Most polygenic risk score (PRS)models have been based on data from populations of European origins (accounting for the majority of the large genomics datasets, e.g. >78% in the UK Biobank and >85% in the GTEx project). Although several large-scale Asian biobanks were initiated (e.g. Japanese, Korean, Han Chinese biobanks), most other Asian countries have little or near-zero genomics data. To implement PRS models for under-represented populations, we explored transfer learning approaches, assuming that information from existing large datasets can compensate for the small sample size that can be feasibly obtained in developing countries, like Vietnam. Here, we benchmark 13 common PRS methods in meta-population strategy (combining individual genotype data from multiple populations) and multi-population strategy (combining summary statistics from multiple populations). Our results highlight the complementarity of different populations and the choice of methods should depend on the target population. Based on these results, we discussed a set of guidelines to help users select the best method for their datasets. We developed a robust and comprehensive software to allow for benchmarking comparisons between methods and proposed a computational framework for improving PRS performance in a dataset with a small sample size. This work is expected to inform the development of genomics applications in under-represented populations. PRSUP framework is available at: https://github.com/BiomedicalMachineLearning/VGP

## Introduction

As of 2021, about 86% of genomics data are from individuals of European descent [1]. The transferability to other ancestries relies on differences in multiple factors including linkage disequilibrium (LD), allele frequencies and genetic architecture [2]. Although Asians account for 23% of the global population, genetic data for Asians are limited. Recently, there is an increased number of studies to identify population-specific causal variants for non-European populations for well-established complex traits such as lipid traits in the Chinese population [3]. Another study used the Japanese biobank, a large resource from genotyping about 200 000 individuals with information on 58 quantitative traits [4]. More Asian population genetics studies are emerging, and more data are being generated using Illumina and Affymetrix arrays. Large Asian biobank data are established but from developed countries such as Japan, Korea, China and India. Meanwhile, genotyping data for developing countries like Vietnam, Indonesia and Thailand are extremely scarce [5]. Vietnam is a developing country with 97 million people and the 15th largest population; however, genetics data for Vietnamese had almost non-existed before the work from the recent 1000 Genome Project [6]. The under-representation, in this work, indicates lack of genomics data in a large population compared with well-studied populations.

Polygenic risk score (PRS) has a great potential to identify and stratify individuals with risk of diseases or prediction of complex traits [7]. It is known that transferability of the underlying model parameters from European to Asian populations is dependent on many factors like LD, allele frequencies, genetic architecture, genetic selection, traits, gene–environment interactions [8]. Consistently, across multiple traits, PRS accuracy was reduced by about $ \sim 37\%$, $ \sim 50\%$, $ \sim 64\%, \sim 78\%$ in for populations with South-Asian, East-Asian and African ancestries, respectively, compared with the predictions for European ancestry (EA) [8, 9]. Current methods optimized for cross-ancestry problems mostly focus on adjusting single nucleotide polymorphisms (SNP) effect sizes in a PRS model by combining summary level data and adjusting SNP effects from different studies (e.g. from European UK Biobank with East Asia). Several studies attempted to incorporate LD, minor allele frequencies (MAF), cross-population correlations of causal SNP effects and heritability to improve accuracy [10]. In general, contemporary studies suggest low transferability, for example a study to generalize PRS scores from EA to Latinos [7].

In this study, we developed a standardized workflow and a scalable software program to assess the transferability of the PRS prediction model across different ancestries so that strategies for improving PRS score for Vietnam using existing data. We evaluated statistical models currently available for trans-ethnicity predictions with the meta-population and multi-population strategy, using both real and simulated data.

**Figure 1:**
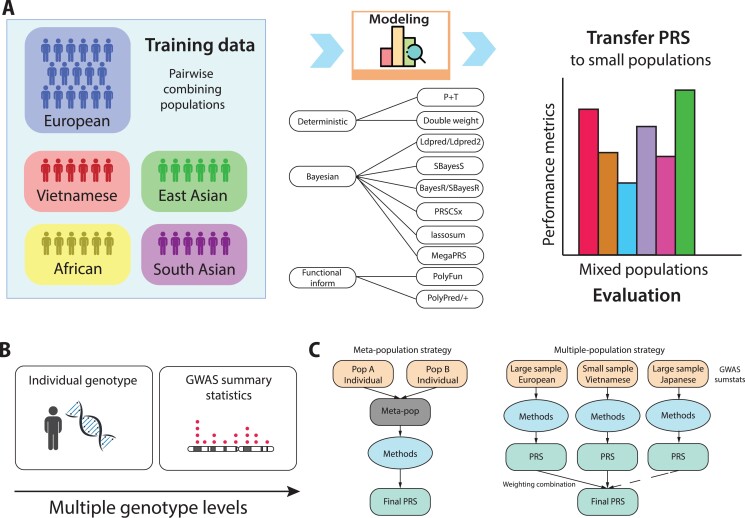
PRSUP benchmarking framework. (**A**) An overview of a generic PRSUP workflow to assess the difference in the performance gained from transferring information across populations to improve PRS for an under-represented population, i.e. the Vietnamese population in this work. Using either simulated or read data from the input of five different populations, three groups of PRS methods (deterministic, Bayesian and functional-inform) were applied to generate the PRS for multiple scenarios. In the last step, models will be transferred to calculate the PRS for the small populations and the performance was compared using common metrics. (**B**) The benchmarking framework can be applied for multiple genotype levels, either simulated or real data, and with or without individual genotyping data. (**C**) Two main strategies to perform benchmarking are meta-population and multiple-population strategies. The meta-population approach combines individual-level genotyping data into a meta-population as the input for PRS methods. The multiple-population strategy performs PRS using GWAS summary data (sumstats) for each population separately and then combines the results into a final PRS score.

## Results

### Selection of 13 trans-ethnicity combinational models for assessing trans-ethnicity PRS models

For benchmarking, we selected 13 commonly used PRS methods representative for three categories, including: fast and deterministic methods with Pruning and Thresholding (PT) [11], double weight (DW) [12]; LD-related methods with LDpred [13] or LDpred2 [14], lassosum [15], SBayesS [16], BayesR or SBayesR [17], MegaPRS [18], PRS-CSx [19] and functionally informed methods with PolyFun [20], PolyPred and PolyPred+ [21] (Figure 1A). A comparison of these methods is shown in Supplementary Figure S1. The PT method is the most simple, yet fast and accurate. In PT, variants are pruned based on LD-clumping and *P*-value thresholding to be included in the PRS calculation. Other methods require LD information and adjust the association of variants from genome-wide association studies (GWAS). Lassosum, LDpred and LDpred2 [13] make use of the LD scores to compute the posterior Single Nucleotide Polymorphisms (SNP) association. SBayesS, BayesR and SBayesR [17] and MegaPRS assume the mixture of distribution of the association coefficients. SBayesR is a version of BayesR, that uses summary-statistics rather than genotype to calculate PRS. PRS-CSx [19] is a recent method developed from PRS-CS that uses LD information from each population and estimates the posterior coefficients using a continuous shrinkage method. PolyFun, PolyPred and PolyPred+ [21] employ functionally informed fine-mapping methods to obtain the posterior association coefficients (Supplementary Figure S1). We developed and evaluated two strategies to integrate methods and datasets to improve PRS performance for an under-represented population, named as meta-population and multiple-population strategies in the individual and summary statistics level (Figure 1B).

### Experimental design for evaluating two combination strategies

For the meta-population strategy, we used genotype inputs at the individual level from two ancestries in the training data set and tested on one of the matched ancestry populations (Figure 1C—left). We hypothesized that the combination increases the heterogeneity and sample size of the training population, thereby resulting in improved performance in GWAS analysis. We implemented two simulated case studies: using only chromosome 22 with seven methods (PT, BayesR, SbayesR, LDpred, PRSCSx, PolyPred and PolyPred+) and using all chromosomes, which were used to calculate PRS using one of the six methods (PT, DW, SbayesR, SbayesS, lassosum and MegaPRS) (Table 1).

**Table 1:** The methods are implemented in each experiment

		P+T	DW	BayesR/SBayesR	SBayesS	ldpred/ldpred2	lassosum	MegaPRS	PRScsx	PolyPred/PolyPred+/PolyFun
Strategies	Meta-population (chr22)	✓		✓		✓			✓	✓
	Meta-population (all chr)	✓	✓	✓			✓	✓		
	Multiple-population	✓	✓	✓	✓	✓				✓

For the multiple-population strategy, we use summary statistics to increase PRS performance by combining weights from each of the two or three populations when optimized by different combinations of eight methods (PT, DW, PolyFun, SbayesR, SbayesS, LDpred2, PolyPred and PolyPred+; note that PolyFun with two population means PolyPred and with three populations means PolyPred+) modelling the PRS (Figure 1C—right;Supplementary Table 1). Combining the sumstats from the largest public database like the UK Biobank, with the sumstats from the training dataset of 1000 Vietnamese, we aimed to improve the prediction accuracy in the target Vietnamese population in the actual dataset of interest (Figure 1C—right). Similarly, we used the Japanese biobank as the additional population to be combined to the Vietnamese sumstats and assessed the PRS performance of the models that combines of the PRS results. We hypothesized that the integrated data of populations with closely related ancestries would improve the signal from common variants that are proportional to the genetic distance between the ancestries and this approach is effective to improve the PRS prediction accuracy. We compared the performance metrics from these methods to derive the most accurate combination.

**Figure 2:**
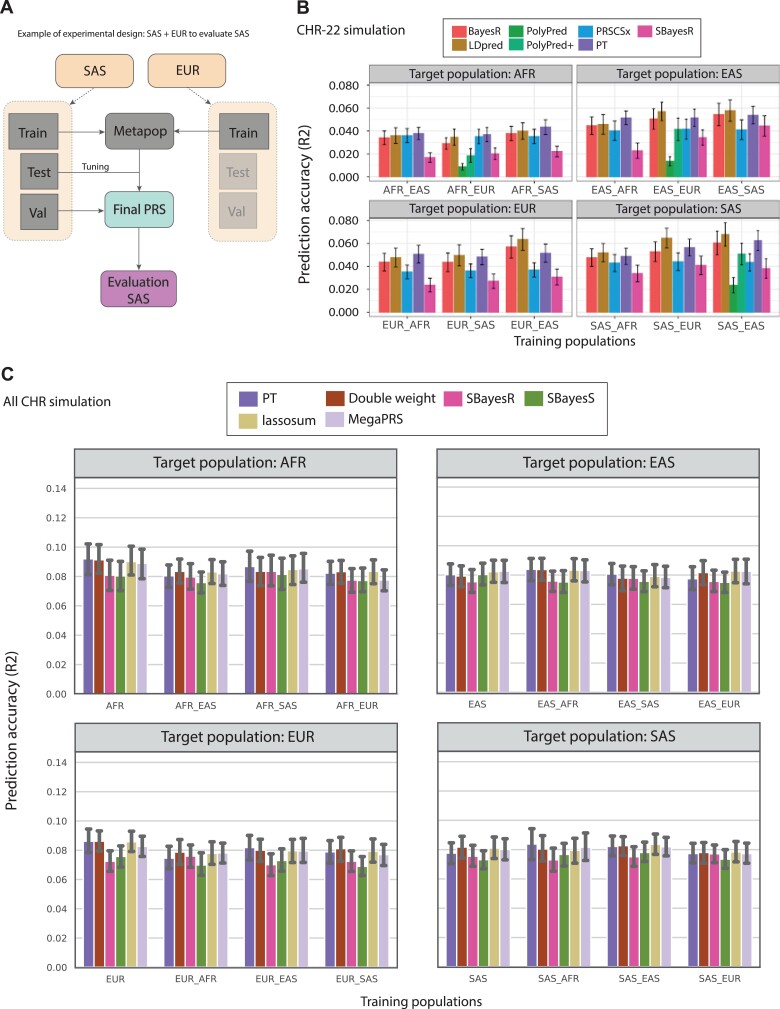
Experimental design and evaluation of the meta-population strategy across four target population AFR, EAS, EUR and SAS. (**A**) An example of experimental design and workflow of meta-population strategy with the combination between SAS and EUR population to assess PRS performances for SAS population (using simulated data). (**B**) The prediction accuracy is estimated as the R2 between the PRS and the simulated phenotype. The phenotype was simulated using 2496 causal SNPs randomly selected from 24 959 SNPs on chromosome 22 from the real genotype in the 1000 Genomes, with effect sizes following a normal distribution $N(0, h2/1000)$. Here we performed seven PRS methods including five methods for all combinations of reference samples (PT, BayesR, SBayesR, LDpred, PRS-CSx) and two methods that leverage the tagging and causal SNP effects of EUR population to predict non-European population (PolyPred and PolyPred+). The PolyPred and PolyPred+ are only performed where EUR is a part of the reference sample. The bars represent the mean R2 across 100 replications of random sampling. The error bars indicate $95\%$ confidence intervals. (**C**) We further simulated phenotype with randomly selected 96 342 SNPs on all chromosomes from the 1000 Genome Project in the same way that we did for the case of using only chromosome 22. We applied PT, DW, SbayesR, SbayesS, lassosum and MegaPRS to model the meta-population PRS. CHR = Chromosome; AFR = African; EUR = European; SAS = South Asian; EAS = East Asian.

### Standardized software for benchmarking

We designed and implemented a scalable software program [PRS for under-represented population(PRSUP)]to incorporate and compare multiple PRS methods (both meta and multiple population strategies), (Supplementary Figure S2). PRSUP is flexible and allows for customization of each model when combining multiple models into one pipeline. PRSUP software helps users find the best method to use for their datasets to make use of transfer learning of information trained from existing data in populations with much more genetics information. The comprehensive meta-population PRS pipeline includes all necessary steps from reading and preprocessing data input, to running PRS methods, generating PRS scores, evaluating different combinations and visualizing results (Supplementary Figure S2).

Specifically, we created a DataProcessor object to consistently centralize the computation of all different datasets used in the benchmarking work. The DataProcessor object contains five input layers and five processed layers.

Input data layers: ‘sumstats’ contains the main summary statistics as the result of the training dataset; ‘test’ contains the genotype of the test dataset; ‘validation’ contains genotypes of the validation dataset (as cross-validation or leave-out validation); ‘phenotype’ contains phenotype of the corresponding test genotype; ‘phenotype_val’ contains phenotype of the corresponding validation genotype. In addition, phenotype files can also have a covariate matrix if it is required in the evaluation process.

Processed data layers: ‘adjusted_ss’ stores the adjusted sumstats path file for each method; ‘prs_validation’ stores the generated PRS scores for the validation dataset; ‘prs_test’ stores the generated PRS scores for the test dataset; ‘tuning’ stores performance metrics from validation data to tune the parameters; and ‘performance’ stores performance metrics of test data. In addition, we provided a set of preprocessing functions for data cleaning, correction and principal component analysis.

### Meta-population—combining individual level genotyping data to employ the overlap in genetic architecture for PRS

We performed simulations based on HAPMAP3 variants that are also present in the Vietnamese 1000 Genome project. We generated two scenarios: randomly selected 10% of causal SNPs from the total of 24 959 from the chromosome 22 and 96 342 SNPs from all chromosomes. We assessed the meta-population approach with 13 commonly used PRS methods including PT, DW, BayesR, SBayesR, SbayesS, LDpred, lassosum, MegaPRS, PRS-CSx, PolyPred and PolyPred+.

We designed a workflow to evaluate the result of the meta-population strategy as exemplified in Figure 2A, where we used the combination of SAS and EUR to model the PRS for the target SAS population. First, we divided each population into three subsets: training, testing and validating subsets. To create meta-populations, we sampled from simulated populations in training/testing/validating sets pairwisely. We merged genotypes (predictors) and phenotype (target) between four population Africa—AFR (AFR_EAS, AFR_EUR, AFR_SAS), East Asia—EAS (EAS_AFR, EAS_EUR, EAS_SAS), Europe—EUR (EUR_AFR, EUR_EAS, AFR_SAS), South Asia—SAS (SAS_AFR, SAS_EAS, SAS_EUR). As a result, twelve pairs of meta-populations were obtained as the baseline, together with four single populations. Each meta-population was used to calculate the PRS for the corresponding target population(e.g. training SAS_EUR to evaluate testing SAS). We first generated the corresponding sumstat and then run the PRS model using the Metapop sumstat using the testing data of the target population, which was used to tune the parameters to obtain the final adjusted sumstat. With that sumstat, we calculated the final PRS with the validating subset to evaluate the performance improvement. The statistical estimate was obtained by running 100 simulated replications. With this design, we aimed to independently assess if there was an improvement in PRS performance when using meta-populations compared with a single population and which methods would be most suitable to achieve the improvement.

For performance assessment, we used the R2 score as the prediction accuracy metric. In the simulated case using only chromosome 22, we observed that the LDpred showed the best prediction accuracy across different target populations (Figure 2B). Consistently, in the target EAS, EUR and SAS, LDpred reached $0.059\pm 0.01$, $0.062\pm 0.01$ and $0.069\pm 0.01$. A deterministic, simple model like PT performed well for the target AFR with $0.042\pm 0.01$. The 2nd top performing method for this meta-population strategy was BayesR, which has slightly lower accuracy compared with LDpred, (0.057 $\pm $ 0.01 in EAS, 0.059 $\pm $ 0.01 in EUR, 0.06 $\pm $ 0.01 in SAS), but outperformed other methods (Figure 2B). In contrast, SBayesR, a model that is based on summary statistics, had the lowest prediction accuracy across different simulation methods (Figure 2B).

In the simulations using all chromosomes, we found that the performance metric was more stable with just just slightly differences against varying combinations of meta-populations and across PRS methods (Figure 2C). PT achieves the highest performance with four meta-populations: AFR-AFR (0.096$\pm $0.01), EAS_AFR-EAS (0.082$\pm $0.008), EUR-EUR (0.085$\pm $0.01) and SAS_EUR-SAS (0.084$\pm $0.01). In contrast, SbayesR and SbayesS had the lowest prediction accuracy. For the AFR_EAS-AFR, EAS_EUR-EAS, EUR_SAS-EUR and SAS-SAS, the R2 scores were 0.075$\pm $0.008, 0.074$\pm $0.009, 0.068$\pm $0.008 and 0.071$\pm $0.007 repetitively (Figure 2C). Notably, the prediction accuracy using all chromosomes was 1.5-2 fold higher compared with using only chromosome 22.

Overall, in the simulations using chromosome 20 only, we observed that the combination for the SAS population always yielded the highest prediction accuracy compared with other combinations, for any target population. The EUR combination had lower scores than the SAS population, but the score is higher compared with other combinations. The combination with the lowest PRS accuracy was for the AFR population data. Comparing the target populations, AFR also had the lowest, whereas the SAS had the highest prediction accuracy. This observation was in line with the meta-population strategy, suggesting different effects on PRS performance when combining individual data from different populations. However, in the all chromosome simulation study, the performance improvement by meta-population compared with the single population was not apparent, except for some slight increase for the combination of EAS_AFR-EAS (PT and DW methods) and SAS_EAS-SAS (PT, MegaPRS and lassosum). Thus, the meta-population strategy that combine individual data (to increase sample size) and assumes a random set of causal SNPs in our analysis did not appear to improve PRS performance. PRSUP meta-population and simulation framework is useful to test more scenarios on the contribution of causal SNPs and on combining suitable populations to improve PRS.

**Figure 3.**
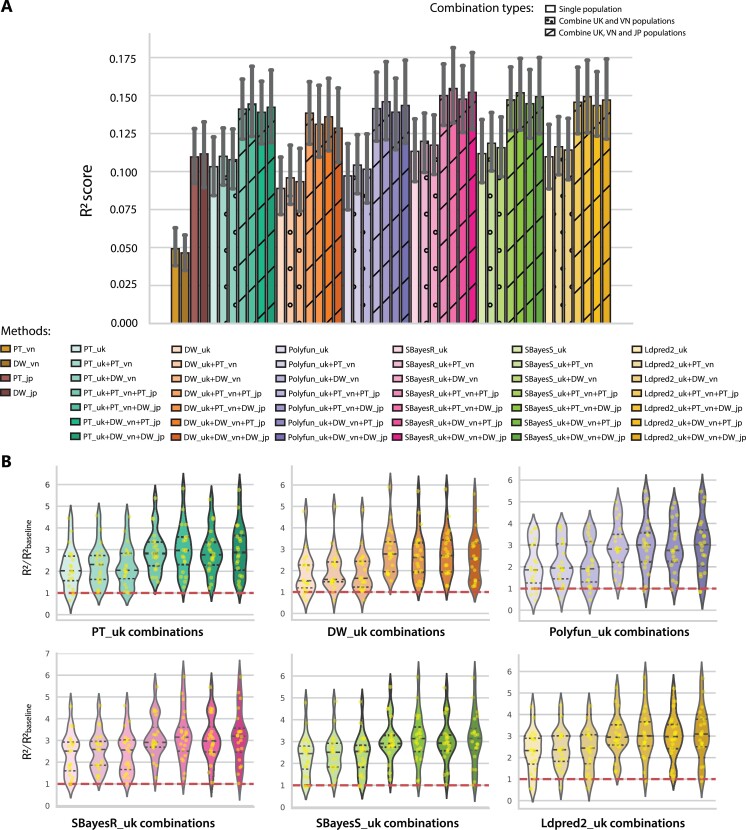
Prediction accuracy of PRS methods using the multiple-population strategy in the target population. (**A**) We used the Vietnamese height phenotype for the PRS model. The prediction accuracy was calculated based on multi-population training. The accuracy was measured as squared correlation (R2) between the true and predicted phenotypes in the testing dataset, averaged across 20-fold cross-validations for the whole genome except for chromosomes X and Y. The error bar indicates the standard deviation of R2 across 20-fold. (**B**) Relative prediction accuracy of single and multi-population PRS using UKB combined with VN and JPN populations, with respect to the baseline models which is the average of PT and DW using 20-fold cross-validation in the Vietnamese population.

### Multiple-population—weighting methods to combine multiple populations summary statistics

In the training phase, we implemented the multiple-population strategy in two ways. One is to combine summary statistics from UK Biobank and VN (1KG project). Another way is to combine the three sources UKB, JP (Japanese biobank) and VN (1KG) to construct the PRS models for the height phenotype of the VN population as the small target population. For the evaluation, we performed the single population PRS models for the UK, JP and VN. For the methods, we used PT, DW, PolyFun, SBayesR, SBayesS and Ldpred2. In total, we obtained results from 20-fold cross-validations (splitting train, test and validation with a 7:1.5:1.5 ratio) from 10 single-population PRS models and 36 multiple-population PRS models.

Figure 3A compares all 46 PRS models for all single populations, combined UK-VN, and combined UK-JP-VN populations. The bar plot shows the average R2 scores from 20-fold cross-validation. compared with the single population $PT\_ uk$ and $DW\_ uk$ the weighting combination models when we added the VN population to the UK population for PT and DW all showed an improvement of R2 score of around 0.005. After adding the JP population, we obtained the results from the UK–JP–VN combination, with a significant increase at about 0.04–0.05 compared with the single UK population or even single VN and JP population. In general, the results showed that combining sumstat of more populations resulted in higher performance in prediction accuracy. We extend the experiment with the body mass index (BMI) phenotype in the 1000 Vietnamese Genome project (Supplementary Figure 3). We observed a consistent pattern compared with the results for the height phenotype. Thus, our analyses strongly suggest that the multiple-population strategy increases the power of the PRS model.

In Figure 3B, we focused on the relative performance of each method and its combinations by comparing the R2 score with the baseline as the average R2 score of single VN population models (PT_vn and DW_vn). For the PT_uk combinations, $PT\_ uk+PT\_ vn+DW\_ jp$, $Polyfun\_ uk+PT\_ vn+DW\_ jp$, $SBayesR\_ uk+PT\_ vn+DW\_ jp$, $SBayesS\_ uk+PT\_ vn+DW\_ jp$ and $Ldpred2+PT\_ vn+DW\_ jp$ have produced higher performance compared with each corresponding single population methods and performed, on average, 3 times better than the baseline models. For the combination of $DW\_ uk$, the $DW\_ uk+PT\_ vn+PT\_ jp$ outperformed other methods. The consistency of $X+PT\_ vn+ DW\_ jp$ ($X$ is any method applied to the UK population) in the R2 score suggested that this combination strategy for trans-ethnic PRS prediction was the most robust. Also, SBayesR is the top performance method that reached 0.156$\pm $0.025 as the highest in $SBayesR\_ uk+PT\_ vn+DW\_ jp$ the PRS models for the height phenotype in the VN population. These results clearly suggest the significant improvement of the multiple-population strategy and the most suitable method to model the PRS in the Vietnamese population.

## Discussion

This work involves assessing cross-population PRS models, with the focus on methods that can enhance PRS performance for populations with little availability of genomics information. This contributes to addressing one of the key challenges in equity in genomics, a critical issue that would lead to healthcare disparities [5, 13]. Specifically, we assessed two independent strategies in PRS modelling approaches to transfer information from existing GWAS studies to the Vietnamese population, utilizing, for the first time, an unique dataset from deep, whole-genome sequencing of 1000 healthy Vietnamese (1KG). We use height as a model trait, using both individual genotyping data (simulated) and summary statistics data (real data).

So far, most existing PRS methods are applied to single populations. We assessed 13 commonly used PRS methods, and noted that only PRS-CSx, PolyPred and PolyPred+ were designed for cross-population prediction. We developed a computational framework to systematically assess the extent of PRS transferability by combining GWAS summary statistics from diverse populations with meta-population and multiple-population strategies. We evaluated seven methods in simulation datasets (meta-population strategy) and 42 combinations of populations from the real summary dataset (multiple-population strategy) with regard to how the additional information from the abundant Caucasian populations and the more closely related Japanese biobank data can add power for the Vietnamese 1KG dataset.

In the meta-population strategy study, we considered the roles of causal variants in improving transferability. Causal variants tend to be shared across populations, while allele frequencies, LD and environmental factors are offer different between populations [8]. Via simulated data analysis, using individual data with considerations of causal SNPs, we showed European and South Asia population pair (meta-population) were the most suitable for transferring the PRS with the meta-population strategy. Future simulation work would include changing genetic architectures, and cross-population genetic correlations (using simulated individual genotyping data). For example, allele frequency would impact the use of causal SNPs if the SNPs are not present in the target population, and similarly if a non-causal, tagged SNP is used in PRS that is in LD with a causal SNP in the discovery dataset but not in the target population. PRS performance was reduced when the genetic distance between the discovery and target cohorts increased. In our simulation study, we generated genotype data, assuming 10% of SNPs as causal, and assessed models that account for causal SNP effects like PolyPred and PolyPred+ and models that do not factor in causal SNPs like PT, LDpred, LDpred2 and PRS-CSx. However, unexpectedly, methods that do not consider causal SNPs, like PT and LDpred, produced higher PRS performance in our simulated datasets. This might be attributed to the use of the infinitesimal model, where all the 24 959/963 426 SNPs are used in the PRS calculation [22].

Multiple-population combining GWAS summary statistics from diverse ethnicities was shown to improve PRS accuracy for under-represented populations [23]. Our results suggested that for under-represented populations, approaches that combine diverse populations tend to produce better performance than approaches that use small-size ancestry-matched data [23]. Using real-world analysis of Vietnamese whole genome sequencing data for height, we found a flexible transfer learning approach using the multiple-population strategy that can consistently improve performance.

## Methods

### Data

#### The 1000 Vietnamese Genomes Project

The 1000 Vietnamese Genomes Project (1KVG) consists of 1008 individuals and nearly 30 million SNPs. The 1KVG dataset is available under agreement at the MASH data portal (https://genome.vinbigdata.org/). We excluded SNPs with MAF < 0.01, Hardy–Weinberg Equilibrium *P*-value < 1e-7, and missingness > 5. We only keep the first SNP if there are duplicated SNPs. We selected only HAPMAP3 variants due to their reliability. These filters result in about 1 million SNPs used in this study.

#### Public GWAS panels

We downloaded the summary statistics of height from 360 000 Europeans in the UK Biobank from Neale’s lab [24] and 159 095 Japanese in the Japan Biobank [25]. The UK Biobank and Neale’s lab contains 28 987 534 and 26 367 797 SNPs, respectively. We filtered out SNPs with MAF < 0.005. The GWAS for the UK Biobank and Japanese Biobank after quality control has 10 832 725 and 9308 743 SNPs, respectively.

### Simulation data

We simulated the phenotype based on the actual genotype data from the Vietnamese Genome Project and 1000 Genomes Project. We randomly selected 10% as causal SNPs to determine the polygenicity of the trait: 2496 HAPMAP3 variants were randomly sampled on chromosome 22 and 96 342 variants on all chromosomes from about 1 million HAPMAP3 variants across different populations. The effect size of these causal SNPs was simulated following the normal distribution with $N(0,\frac{h^{2}}{M})$ where $h^{2} = 0.5$ was the heritability of the causal SNPs and $M$ was the number of selected causal variants. The residual phenotype was simulated as $N(0,1-h^{2})$ such that the total phenotypic variance was equal to $1$. For each population, we generated 100 replications to unbiased evaluate the meta-population strategy.

#### PT

The PT method utilizes informed LD-pruning and *P*-value thresholding on marginal effects. We calculated this PRS using PRSice-2 with default parameters [26], we selected SNPs with *P*-values ($P_{T}$) ¡1e-8, 1e-7, 1e-6, 1e-5, 3e-5, 1e-4, 3e-4, 0.001, 0.003, 0.01, 0.03, 0.1, 0.3, 1, pairwise LD r2 ¡0.1 within a physical distance of 250 kb. The PRS is then estimated with the selected SNPs. The PT values with the highest prediction accuracy were selected for the final PRS.

#### Double weight

DW is another deterministic method that uses a hard threshold to choose the list of SNPs for PRS. The idea is to systematically pick up a set of SNPs by estimated *P*-values lower than a threshold with effect overestimated by chance. Then the betas will be a biased estimate for the true weight.

$$\begin{align*} PRS = \sum_{i=1}^{k}\hat{\pi} (X_{i})\hat{\beta }X_{i} \end{align*}$$

Where $\hat{\pi }$ is the weighting for each SNP in the Z top number of SNPs set up by threshold. Within the supplementary states, the sample of values for each SNP should be formed from a normal distribution specified by $N(\hat{\beta }, \hat{SE}^{2})$

#### LDpred and LDpred-inf

The LDpred method estimates PRS based on the inferred posterior effect size from the GWAS panel while considering for LD from an independent reference panel from the same population with the point-normal prior on the SNP effect sizes. The assumed point-normal prior on the SNP effect sizes $\beta _{j}$

$$\begin{align*} \beta_{j} \approx \begin{cases} N(0,\frac{h^{2}_{g}}{\pi M}) & \text{with probability }\pi\\ 0 & \text{with probability 1-}\pi, \end{cases} \end{align*}$$

where $h_{g}^{2}$ is the SNP heritability, $M$ and is the total number of SNPs and proportion of causal variants, respectively. LDpred utilizes the MCMC sampling to estimate the posterior mean $j$ given the marginal effect from GWAS and LD from the reference panel. Here we used the default values with 1e-5, 3e-5, 1e-4, 3e-4, 0.001, 0.003, 0.01, 0.03, 0.1, 0.3, 1. The $\pi $ with the highest prediction accuracy was selected to estimate the PRS.

With $\pi =1$, LDpred-inf is the version of LDpred in the scenario where all variants are assumed to be causal. With the infinitesimal model, the posterior mean effect sizes can be estimated:

$$\begin{align*} E\left [ \hat{\beta}_{l},D_{l} \right ] = \left ( D_{l} + \frac{M}{Nh_{g}^{2}} I \right )^{-1} \hat{\beta_{l}}, \end{align*}$$

where $\beta _{l}$ is the marginal least squares effect size, $D_{l}$ is the LD matrix that can be estimated from the external reference panel, $I$ is an identity matrix and the heritability, $h_{g}^{2}$, is assumed to be small such that $1-h2 \approx 1$.

#### LDpred2

LDpred2 has two new extensions compared with the LDpred model. It addresses the issue of instability in long-range LD regions and computational efficiency. First, LDpred2 provided the extension that consists of assuming a point-normal mixture distribution for effect sizes, where only a proportion of causal variants p contributes to the SNP heritability h2. Thus, this gives the LDpred2-auto as a method free of hyper-parameters and can be applied directly to data without the need of a validation dataset for tuning parameters. Second, the LDpred2 model enables using a third hyper-parameter in the grid mode that aims at size estimating the sparse effect.

#### BayesR and SBayesR

BayesR and SBayesR employ Bayesian multiple regression to rescale the marginal SNP effect. These methods assume that the standardized SNP effects follow a mixture of multiple zero-mean normal distribution:

$$\begin{align*} \beta_{j} | \pi \sigma^{2}_{\beta} = \begin{cases} 0 & {\textrm{with probability}\ \pi_{1}}\\ \sim N(0,\gamma_{2} \sigma_{\beta}^{2} ) & \text{with probability }\pi_{2} \\ \sim N(0,\gamma_{C} \sigma_{\beta}^{2} ) & \text{with probability }1-\sum_{c=1}^{C-1} \pi_{c}, \end{cases} \end{align*}$$

where C is the number of components in the finite mixture model, $\gamma $ is the constrain to rescale the total variance from marginal effects for each distribution. In terms of the input, BayesR uses genotype-level data, whereas SBayesR makes use of the marginal effects estimated from the summary statistics. With BayesR, we used $C=4$ and $\gamma $ = ($\gamma _{1}$,$\gamma _{2}$,$\gamma _{3}$,$\gamma _{4}$) = (0, 0.0001, 0.001, 0.01) as proposed by Moser *et al*. For SBayesR, we used $C=4$ with $\gamma $ = ($\gamma _{1}$,$\gamma _{2}$,$\gamma _{3}$,$\gamma _{4}$)=(0, 0.01, 0.1, 1). The LD matrix is calculated with GCTB software with a randomly selected set of 10K UK Biobank individuals.

#### SBayesS

The SBayesS model exploit the effect of natural selection on SNPs, which is illustrated through the MAF. These methods assume that the SNP effect j has a hierarchical mixture prior:

$$\begin{align*} \beta_{j} \sim N(0,[2p_{j}(1-p_{j})]^{S \sigma_{\beta}^{2}})\pi + \phi (1- \pi), \end{align*}$$

where $p_{j}$ is the MAF, $\pi $ is the proportion of SNPs with non-zero effects indicating the polygenicity of the trait. The association between variance of SNP effects and MAF is modeled by S, which follows a normal distribution $N(0,\sigma _{s}^{2})$. We use the starting default S = 0.

#### Lassosum

Lassosum does not rely on computing SNP effect sizes that would be produced with full genetic information. This method is inspired by the lasso regression, in a more heuristic or goal-oriented setting. Lassosum not only has high accuracy but is also very fast.

#### MegaPRS

MegaPRS contains four models, LDAK-Lasso-SS, LDAK-Ridge-SS, LDAK-Bolt-SS and LDAK-BayesR-SS. In this paper, we chose the LDAK-BayesR-SS for modeling the PRS with three steps. Step 1, the reference panel is used to estimate correlations between all pairs of SNPs. Step 2, MegaPRS constructs pairs of PRS models using training sumstats (subset), followed by using the full summastats. Step 3, MegaPRS uses the subset of test sumstat to identify the most accurate training models. The final result is the adjusted effect sizes for the selected model.

#### PRS-CSx

The PRS-CSx method exploits the correlation between genetic effects while considering the allele frequency and the LD information in each population to improve the association signal. For SNP j in the population k, the effect size is modeled as a global–local scale mixture of normal distributions:

$$\begin{align*} \beta_{jk} \sim N\left(0,\frac{\sigma_{k}^{2}}{N_{k}} \phi \psi_{j}\right) \\ \psi_{j} \sim Gamma(a,\delta_{j} ) \\ \phi \sim Gamma(b,1), \end{align*}$$

where $\phi $ is the global shrinkage parameter to model the sparseness between all SNPs the genetic information, $\psi _{j}$ is the local shrinkage parameter for marginal association, $\sigma _{k}^{2}$ is the variance of non-genetic effects, $N_{k}$ is the number of individuals. The local shrinkage parameter follows a gamma–gamma hierarchical prior.

#### Polyfun, PolyPred and PolyPred+

PolyPred makes use of the European training data to improve the cross-ancestry PRS by combining two predictors: (1) the existing BOLT-LMM to estimate the tagging effect and (2) functionally informed fine-mapping to estimate causal effects. Additionally, when there is a large sample size of non-European population, PolyPred+ incorporates these non-European populations considering differences in MAF and causal effect sizes. The effect of SNP j is defined as:

$$\begin{align*} \hat{\beta}^{PolyPred}_j = w^{PolyFun-pred} \hat{\beta}^{PolyFun-pred}_j + w^{BOLD-LMM} \hat{\beta}^{BOLT-LMM}, \end{align*}$$

where $\hat{\beta }^{PolyFun-pred}_j$ is the estimated posterior mean causal effect size of SNP j using PolyFun-pred on the European training data, $\hat{\beta }^{BOLT-LMM}$ is the posterior mean tagging effect size of SNP j. The mixing weight $w^{PolyFun-pred}$ and $w^{BOLD-LMM}$ is estimated by regression the phenotype on the effect size $\hat{\beta }^{PolyFun-pred}_j$ and $\hat{\beta }^{BOLT-LMM}$, respectively.

The estimated mixing weights are calculated by the non-negative least squares from training the phenotype of non-European on the PRS estimated from each summary statistics.

In addition, PolyPred+ integrates the tagging effect from the non-European population to PolyPred:

where $\hat{\beta }^{BOLT-LMM-nonEur}_j$ is the tagging effect size from the non-European population. The mixing weight $w^{BOLD-LMM-nonEur}$ is estimated via non-negative least square estimation.

### Linear combination of weighting factors from multiple predictors

Inspired by PolyPred and PolyPred+, we extend the approach of linear combining two predictors to multiple predictors and the combination is flexible to use any individual predictors (multiple population strategy). The function computes linear combinations of the estimated effect sizes of each constituent predictor:

$$\begin{align*} \hat{\beta} ^{Combination} = \sum_{j} w^{j} \hat{\beta_{i}^{J}}, \end{align*}$$

where $i$ indexes SNPs, $j$ indexes the constituent predictors (method $j$). Per-allele effect size of SNP $i$, $w^{j}$ are method-specific weights, and $\hat{\beta _{i}^{j}}$ is the per-allele effect size of SNP $i$ for method $j$. Predicted phenotypes are computed by applying effect sizes to target genotypes:

$$\begin{align*} \hat{y} = \sum_{i} x_{i} \hat{\beta_{i}}^{Combination}. \end{align*}$$

**Key Points**

- The majority of genome-wide association studies have been performed in large populations of Caucasian origin, leading to inequity in genomics. Therefore, the vast potential clinical applications of polygenic risk scores (PRS), which are constructed based on GWAS, would lead to healthcare disparities.
- To tackle this challenge, we propose a scalable and customizable framework PRSUP for benchmarking transfer learning approaches in PRSs. These approaches are to address the problem of implementing the combination models in populations with under-represented genomic data. We present detailed analysis using the Vietnamese population, but the PRSUP approach can be applicable to other under-represented populations.
- PRSUP combined and benchmarked existing PRS methods in a meta-population strategy (combining individual data from multiple populations) and a multi-population strategy (combining PRS results from multiple populations).
- PRSUP meta-population strategy with individual level simulated genotypes and taking into account the roles of causal variants. PRSUP showed the improvement from combining European and South Asia population models using LDpred.
- PRSUP multiple-population strategy uses summary statistic data for under-represented populations and combines diverse populations to produce better performance than approaches that use only small-size ancestry-matched (Vietnamese) data.

## Data availability

The data and code are publicly available via: https://github.com/BiomedicalMachineLearning/VGP.

## Supplementary Material

Revised_supplementary_information_bbac459
