# Supplementary material for: Assessing polygenic risk score models for applications in populations with under-represented genomics data: an example of Vietnam
Source: Brief Bioinform. 2022 Nov 2;23(6):bbac459. doi: 10.1093/bib/bbac459 (PMC9677487; doi:10.1093/bib/bbac459)
Supplement: Revised_supplementary_information_bbac459 [file revised_supplementary_information_bbac459.pdf]

Supplementary Information

Authors: Duy Pham, Buu Truong, Khai Tran, Guiyan Ni, Dat Nguyen, Nam S. Vo, Quan Nguyen

Type	Method	Category	Genetic architecture	LD reference	Adjust causal effect sizes	Optimization method	Prior (Bayesian)	Heritability
**Single population**	P+T	Deterministic	No information	Not required	None	None	None	None
	Double weight	Deterministic	No information	Not required	Winner's curse	None	None	Partial correction of some common biases may lead to a reduced amount of "missing heritability"
	ldpred	Bayesian	Wide range of genetic architectures	Required (Initially/widely used when accounting for LD, underpowered for less polygenic traits)	Point-normal prior, p proportion of causal SNPs	None	Point-normal mixture	Prediction accuracy converges to the heritability explained by the SNPs as sample size grows
	ldpred-inf	Bayesian	Wide range of genetic architectures	Required	Infinitesimal model	None	Point-normal mixture	Prediction accuracy converges to the heritability explained by the SNPs as sample size grows
	ldpred2	Bayesian	Assumes all SNPs have non-zero contribution of the phenotype	Required	Point-normal prior, p proportion of causal SNPs	None	Point-normal mixture	Automatically calculation
	SbayesS	Bayesian	Focus on pervasive signatures of negative selection in the genetic architecture	Required	Mixture of three normal distributions with small, medium and large variances:	None	Point-normal mixture	SNP-based heritability estimation using MCMC
	bayesR	Bayesian	Contained very large genetic effects and a polygenic background	Required		None	Point-normal mixture	SNP-based heritability estimation using MCMC
	sbayesR	Bayesian	Contained very large genetic effects and a polygenic background	Required	4 mixture normal distributions	None	Point-normal mixture	SNP-based heritability estimation using MCMC
	PRScs	Bayesian	Wide range of genetic architectures	Required	The continuous shrinkage priors	None	Continuous shrinkage	Heritability is estimated based on assumed population and sample prevalences
	lassosum	Deterministic	No information	Required	Penalized regression (LASSO/Elastic Net)	None	None	No information
	MegaPRS	Bayesian	No information	Optional	Construction based on other methods	Grid search	Use the same prior distribution forms as the existing tools	Worked in trait with high heritability
	Winner's curse	Deterministic	No information	Not required	Winner's curse	None	None	if only independent SNPs are analyzed, use of a subset of SNPs similarly enriched for heritability is expected to yield much higher improvement in the performance of the model
	JAMPred	Bayesian	No information	Required	Hierarchical normal prior	None	Hierarchical normal prior	Treating sparsity and local heritability as random quantities rather than fixed hyperparameters
	Tweedie	Bayesian	Wide range of genetic architectures	Not required	Winner's curse	None	Flexible choice of prior	Worked with wide range of heritability
	SBLUP	Deterministic	Wide range of genetic architectures	Required	Kinship among individuals	None		Worked in trait with high heritability
**Multiple population**	Polypred	Bayesian	Model functional architectures	Required (from large panel)	Ensemble (causal SNPs)	NNLS	Mixture-of-normals	Estimating per-SNP heritabilities for SNPs
	PolyPred+	Bayesian	Model functional architectures	Required (from large panel)	Ensemble (causal SNPs)	NNLS	Mixture-of-normals	Estimating per-SNP heritabilities for SNPs
	PRScsx	Bayesian	Wide range of genetic architectures	Required	Gamma-gamma hierarchical prior	Linear regression (polynomial)	Shared continuous shrinkage	Heritability is estimated based on assumed population and sample prevalences
**Functional leveraging**	PolyFun	Bayesian	Model functional architectures	Required	Fine mapping	None	Based on SuSiE or FINEMAP.	Estimating per-SNP heritabilities for SNPs

Supplementary 1. Survey about theoretical behind of nineteen methods. We include their charateristics of category, genetic architecture, LD reference use, way to adjust the effect sizes, optimization methods, prior distribution and heritability. From all of that, we chose thirteen most suitable methods to implement the experiment of the main paper

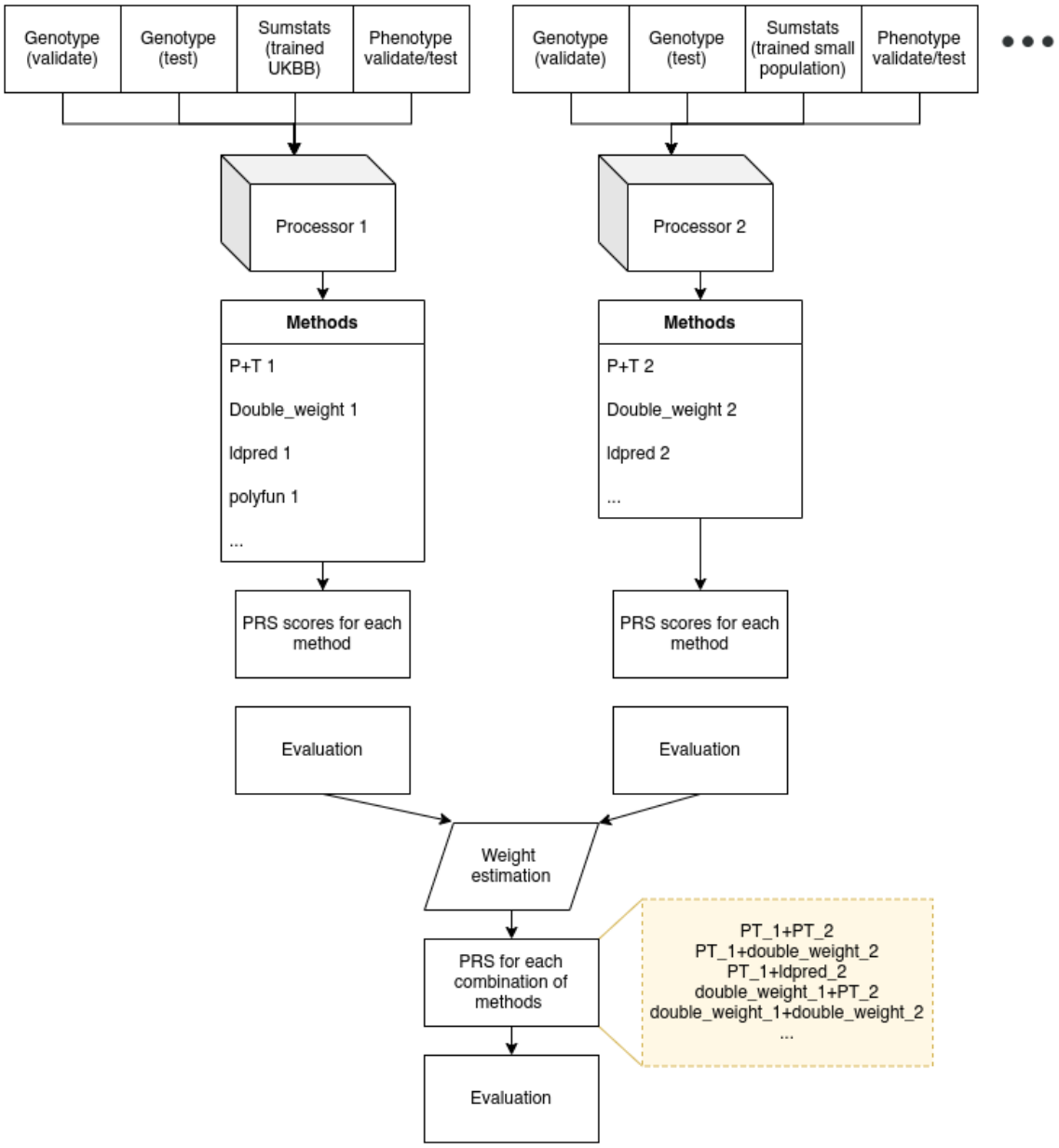

Supplementary 2. Workflow of multiple-population strategy that implemented in our software. Started from a set of sumstats(train), genotype (test and validate) and phenotype (test and validate) for each population. After that, the PRSs are generated for all the methods then combine it by weight estimation. Finally, we are able to evaluate the modelled PRS to assess their performance.

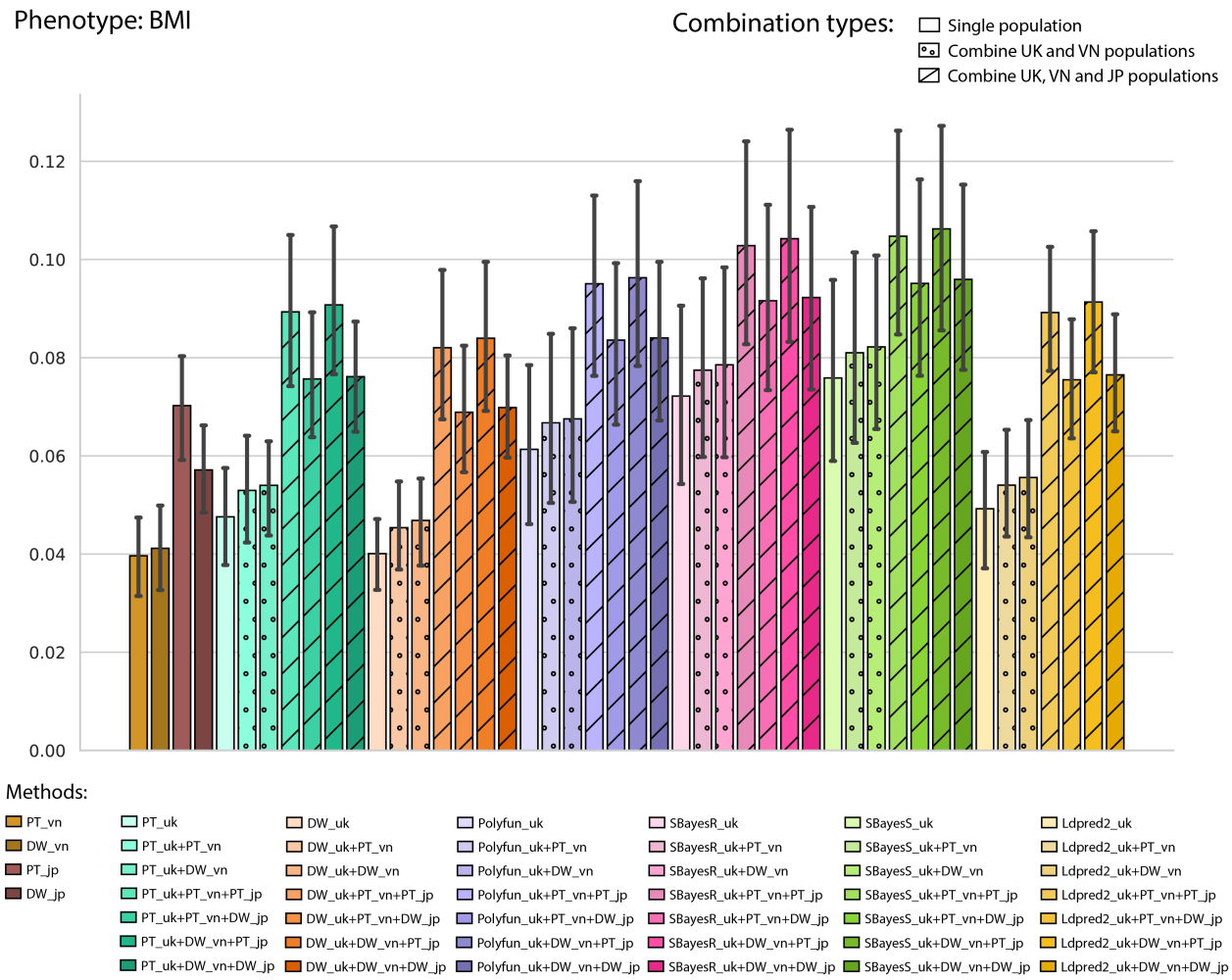

Supplementary 3. Prediction accuracy of PRS methods using the multiple-population strategy in the target population. BMI phenotype data of the Vietnamese population was used for the PRS model and the prediction accuracy was calculated based on multi-population training. The accuracy was measured as squared correlation (R^2) between the true and predicted phenotypes in the testing dataset, averaged across 20-fold cross-validations for the whole genomes except for chromosomes X and Y. The error bar indicates the standard deviation of R2 across 20-fold.

Multiple_population_tutorial

August 23, 2022

1 Multiple populations strategy for transfer PRS tutorial

This tutorial show step by step how to run the transprs framework using multiple population strategy to transfer the PRS model from UKBB (large number of samples) and JPN (large number of samples) to Vietnamese (VN) population (small number of samples)

1.0.1 Loading library

[1]:
```
import transprs as tprs
```

1.0.2 Prepare the input data

In the `tutorials/data` folder that download from here:

https://drive.google.com/file/d/1ZsbfabvwnssMiji6ECDieg1C7mJNhvYm/view?usp=sharing

, you should have:

- Test or target individual genotypes: `test_demo.bed/bim/fam`
- Test phenotype: `test_demo.phenotype`
- Validation individual genotypes: `val_demo.bed/bim/fam`
- Validation phenotype: `validation_demo.phenotype`
- Summary statistic files: `UKBB_height_sumstats.ss` from UKBB, `VN_train_sumstats.ss` from VN and `JPN_height_sumstat.ss` from JPN data

1.0.3 Reading input data

We create a DataProcessor object that manage all the related data and will be used to process and interactive with PRS methods, validation and visualization

[2]:
```
processor_UKBB = tprs.read_input(prefix_test="tutorials/data/test_demo",
                 test_phenotype="tutorials/data/test_demo.phenotype",
                 sumstats_path="tutorials/data/UKBB_height_sumstats.ss",
                 prefix_validation="tutorials/data/val_demo",
                 validation_phenotype="tutorials/data/validation_demo.phenotype",
                 workdir="workdir_UKBB_tutorial")
```

```
Reading the Test genotype...

Mapping files: 100%|| 3/3
[00:00<00:00, 82.54it/s]

Reading the Validation genotype...

Mapping files: 100%|| 3/3
[00:00<00:00, 91.02it/s]

Phenotype stored in .phenotype
Phenotype stored in .phenotype_val
```

1.0.4 Preprocessing

In the preprocessing, we included: cleaning SNPs, flip reverse, compute PCA

[3]:
```
tprs.Preprocessing(processor_UKBB, n_components=6)
```

```
PLINK v1.90b6.21 64-bit (19 Oct 2020)          www.cog-genomics.org/plink/1.9/
(C) 2005-2020 Shaun Purcell, Christopher Chang   GNU General Public License v3
Logging to tmp.log.
Options in effect:
--bfile tmp
--indep-pairwise 200 50 0.25
--out tmp

31807 MB RAM detected; reserving 15903 MB for main workspace.
22331 variants loaded from .bim file.
257 people (0 males, 0 females, 257 ambiguous) loaded from .fam.
Ambiguous sex IDs written to tmp.nosex .
Using 1 thread (no multithreaded calculations invoked).
Before main variant filters, 257 founders and 0 nonfounders present.
Calculating allele frequencies... 10111213141516171819202122232425262728293031 32
33343536373839404142434445464748495051525354555657585960616263646566676869707172
73747576777879808182838485868788899091929394959697989 done.
22331 variants and 257 people pass filters and QC.
Note: No phenotypes present.
Pruned 9950 variants from chromosome 21, leaving 1559.
Pruned 9120 variants from chromosome 22, leaving 1702.
Pruning complete.  19070 of 22331 variants removed.
Marker lists written to tmp.prune.in and tmp.prune.out .
PLINK v1.90b6.21 64-bit (19 Oct 2020)          www.cog-genomics.org/plink/1.9/
(C) 2005-2020 Shaun Purcell, Christopher Chang   GNU General Public License v3
Logging to tmp.log.
Options in effect:
--bfile tmp
--extract tmp.prune.in
--out tmp
--pca 6

31807 MB RAM detected; reserving 15903 MB for main workspace.
22331 variants loaded from .bim file.
257 people (0 males, 0 females, 257 ambiguous) loaded from .fam.
Ambiguous sex IDs written to tmp.nosex .
--extract: 3261 variants remaining.
Using up to 11 threads (change this with --threads).
Before main variant filters, 257 founders and 0 nonfounders present.
Calculating allele frequencies... 10111213141516171819202122232425262728293031 32
33343536373839404142434445464748495051525354555657585960616263646566676869707172
73747576777879808182838485868788899091929394959697989 done.
3261 variants and 257 people pass filters and QC.
Note: No phenotypes present.
Relationship matrix calculation complete.
--pca: Results saved to tmp.eigenval and tmp.eigenvec .
PCA result is stored in .phenotype
PLINK v1.90b6.21 64-bit (19 Oct 2020)          www.cog-genomics.org/plink/1.9/
(C) 2005-2020 Shaun Purcell, Christopher Chang   GNU General Public License v3
```

```
Logging to tmp.log.
Options in effect:
--bfile tmp
--indep-pairwise 200 50 0.25
--out tmp

31807 MB RAM detected; reserving 15903 MB for main workspace.
22331 variants loaded from .bim file.
258 people (0 males, 0 females, 258 ambiguous) loaded from .fam.
Ambiguous sex IDs written to tmp.nosex .
Using 1 thread (no multithreaded calculations invoked).
Before main variant filters, 258 founders and 0 nonfounders present.
Calculating allele frequencies... 10111213141516171819202122232425262728293031323334353637383940414243444546474849505152535455565758596061626364656667686970717273747576777879808182838485868788899091929394959697989 done.
22331 variants and 258 people pass filters and QC.
Note: No phenotypes present.
Pruned 9958 variants from chromosome 21, leaving 1551.
Pruned 9129 variants from chromosome 22, leaving 1693.
Pruning complete.  19087 of 22331 variants removed.
Marker lists written to tmp.prune.in and tmp.prune.out .
PLINK v1.90b6.21 64-bit (19 Oct 2020)          www.cog-genomics.org/plink/1.9/
(C) 2005-2020 Shaun Purcell, Christopher Chang   GNU General Public License v3
Logging to tmp.log.
Options in effect:
--bfile tmp
--extract tmp.prune.in
--out tmp
--pca 6

31807 MB RAM detected; reserving 15903 MB for main workspace.
22331 variants loaded from .bim file.
258 people (0 males, 0 females, 258 ambiguous) loaded from .fam.
Ambiguous sex IDs written to tmp.nosex .
--extract: 3244 variants remaining.
Using up to 11 threads (change this with --threads).
Before main variant filters, 258 founders and 0 nonfounders present.
Calculating allele frequencies... 10111213141516171819202122232425262728293031323334353637383940414243444546474849505152535455565758596061626364656667686970717273747576777879808182838485868788899091929394959697989 done.
3244 variants and 258 people pass filters and QC.
Note: No phenotypes present.
Relationship matrix calculation complete.
--pca: Results saved to tmp.eigenval and tmp.eigenvec .
PCA result is stored in .phenotype
Created folder workdir_UKBB_tutorial for working directory
Stored sumstats workdir_UKBB_tutorial/preprocessed_sumstats in working directory
Stored test genotype workdir_UKBB_tutorial/preprocessed_test in working
directory
Stored validation genotype workdir_UKBB_tutorial/preprocessed_test in working
directory
Stored test genotype workdir_UKBB_tutorial/preprocessed_test in working
directory
```

1.0.5 Running PRS models

We provided a set of common PRS models to use to generate the basic results. They will be combined later.

Current support PRS models: - P+T or Clumping: `tprs.methods.clumping` - Double weight: `tprs.methods.double_weight` - LDpred: `tprs.methods.ldpred` - LDpred2: `tprs.methods.ldpred2` - polyfun: `tprs.methods.polyfun` - SBayesS: `tprs.methods.SBayesS` - SBayesR: `tprs.methods.SBayesR` - PRSCSx: `tprs.methods.multipop.prscsx`

In this tutorial, we will only run the P+T/clumping and Double weight methods for each population.

For each model, there are 5 steps: - 1. Run the method - 2. Generate PRS for validation data - 3. Evaluate with the validation data - 4. Generate PRS for test data - 5. Evaludate with the test data

P+T/clumping model for UKBB

[4]:
```
tprs.methods.clumping(processor_UKBB)

tprs.scoring.generate_prs(processor_UKBB,method="clumping")

tprs.metrics.coef_squared_evaluation(processor_UKBB,
                                     method="clumping",
                                     trait_col="Height",
                                     prs_col="SCORESUM",
                                    )

tprs.scoring.generate_prs(processor_UKBB,method="clumping",validate=False)

tprs.metrics.coef_squared_evaluation(processor_UKBB,
                                     method="clumping",
                                     trait_col="Height",
                                     prs_col="SCORESUM",
                                    validate=False)
```

```
Clumping is running...
PLINK v1.90b6.21 64-bit (19 Oct 2020)          www.cog-genomics.org/plink/1.9/
(C) 2005-2020 Shaun Purcell, Christopher Chang   GNU General Public License v3
Logging to tmp_out.log.
Options in effect:
--bfile workdir_UKBB_tutorial/preprocessed_validation
--clump workdir_UKBB_tutorial/preprocessed_sumstats
--clump-field P
--clump-kb 250
--clump-p1 1
--clump-r2 0.5
--clump-snp-field SNP
--out tmp_out

31807 MB RAM detected; reserving 15903 MB for main workspace.
22331 variants loaded from .bim file.
258 people (0 males, 0 females, 258 ambiguous) loaded from .fam.
Ambiguous sex IDs written to tmp_out.nosex .
Using 1 thread (no multithreaded calculations invoked).
Before main variant filters, 258 founders and 0 nonfounders present.
Calculating allele frequencies... 10111213141516171819202122232425262728293031323334353637383940414243444546474849505152535455565758596061626364656667686970717273747576777879808182838485868788899091929394959697989 done.
22331 variants and 258 people pass filters and QC.
```

```
Note: No phenotypes present.
--clump: 4743 clumps formed from 14365 top variants.
Results written to tmp_out.clumped .
Done clumping!
The clumping result stores in .adjusted_ss['clumping']!
--- Done in 0:00:00 ---
Extracting adjusted sumstats from clumping method...
Generating PRS...
PLINK v1.90b6.21 64-bit (19 Oct 2020)          www.cog-genomics.org/plink/1.9/
(C) 2005-2020 Shaun Purcell, Christopher Chang   GNU General Public License v3
Logging to tmp_results.log.
Options in effect:
  --bfile workdir_UKBB_tutorial/preprocessed_validation
  --out tmp_results
  --q-score-range tmp_range_list tmp_SNP.pvalue
  --score workdir_UKBB_tutorial/adjusted_sumstats_clumping 3 4 9 header sum

31807 MB RAM detected; reserving 15903 MB for main workspace.
22331 variants loaded from .bim file.
258 people (0 males, 0 females, 258 ambiguous) loaded from .fam.
Ambiguous sex IDs written to tmp_results.nosex .
Using 1 thread (no multithreaded calculations invoked).
Before main variant filters, 258 founders and 0 nonfounders present.
Calculating allele frequencies... 10111213141516171819202122232425262728293031323334353637383940414243444546474849505152535455565758596061626364656667686970717273747576777879808182838485868788899091929394959697989 done.
22331 variants and 258 people pass filters and QC.
Note: No phenotypes present.
--score: 4743 valid predictors loaded.
--score: 17 ranges processed.
Results written to tmp_results.*.profile.
PRS is generated!
The PRS result stores in .prs_validation['clumping']!
--- Done in 0:00:00 ---
The best fit p-value is 0.00000001
The best fit result is stored in
processor.prs_validation['clumping']['best_fit']
The best fit result is stored in processor.tuning['clumping']['coef_squared']
Extracting adjusted sumstats from clumping method...
Generating PRS...
PLINK v1.90b6.21 64-bit (19 Oct 2020)          www.cog-genomics.org/plink/1.9/
(C) 2005-2020 Shaun Purcell, Christopher Chang   GNU General Public License v3
Logging to tmp_results.log.
Options in effect:
  --bfile workdir_UKBB_tutorial/preprocessed_test
  --out tmp_results
  --q-score-range tmp_range_list tmp_SNP.pvalue
  --score workdir_UKBB_tutorial/adjusted_sumstats_clumping 3 4 9 header sum

31807 MB RAM detected; reserving 15903 MB for main workspace.
22331 variants loaded from .bim file.
257 people (0 males, 0 females, 257 ambiguous) loaded from .fam.
Ambiguous sex IDs written to tmp_results.nosex .
Using 1 thread (no multithreaded calculations invoked).
```

```
Before main variant filters, 257 founders and 0 nonfounders present.
Calculating allele frequencies... 10111213141516171819202122232425262728293031323334353637383940414243444546474849505152535455565758596061626364656667686970717273747576777879808182838485868788899091929394959697989 done.
22331 variants and 257 people pass filters and QC.
Note: No phenotypes present.
--score: 4743 valid predictors loaded.
--score: 1 range processed.
Results written to tmp_results.*.profile.
PRS is generated!
The PRS result stores in .prs_test['clumping']!
--- Done in 0:00:00 ---
The best fit p-value is 0.00000001
The best fit result is stored in processor.prs_test['clumping']['best_fit']
The best fit result is stored in
processor.performance['clumping']['coef_squared']

Warning: 1 line skipped in --q-score-range data file.
Warning: 1 line skipped in --q-score-range data file.
```

Double weight model for UKBB

[5]:
```
tprs.methods.double_weight(processor_UKBB)

tprs.scoring.generate_prs(processor_UKBB,method="double_weight")

tprs.metrics.coef_squared_evaluation(processor_UKBB,
                                     method="double_weight",
                                     trait_col="Height",
                                     prs_col="SCORESUM",
                                    )

tprs.scoring.generate_prs(processor_UKBB,method="double_weight",validate=False)

tprs.metrics.coef_squared_evaluation(processor_UKBB,
                                     method="double_weight",
                                     trait_col="Height",
                                     prs_col="SCORESUM",
                                    validate=False)
```

```
Double weight method is running...
[1] "Adjusted BETA is done!"
Done Double weight!
The double weight result stores in .adjusted_ss['double_weight']!
--- Done in 0:00:01 ---
Extracting adjusted sumstats from double_weight method...
Generating PRS...
PLINK v1.90b6.21 64-bit (19 Oct 2020)          www.cog-genomics.org/plink/1.9/
(C) 2005-2020 Shaun Purcell, Christopher Chang   GNU General Public License v3
Logging to tmp_results.log.
Options in effect:
  --bfile workdir_UKBB_tutorial/preprocessed_validation
  --out tmp_results
  --q-score-range tmp_range_list tmp_SNP.pvalue
  --score workdir_UKBB_tutorial/adjusted_sumstats_double_weight 3 4 9 header sum
```

```
31807 MB RAM detected; reserving 15903 MB for main workspace.
22331 variants loaded from .bim file.
258 people (0 males, 0 females, 258 ambiguous) loaded from .fam.
Ambiguous sex IDs written to tmp_results.nosex .
Using 1 thread (no multithreaded calculations invoked).
Before main variant filters, 258 founders and 0 nonfounders present.
Calculating allele frequencies... 10111213141516171819202122232425262728293031 32
33343536373839404142434445464748495051525354555657585960616263646566676869707172
73747576777879808182838485868788899091929394959697989 done.
22331 variants and 258 people pass filters and QC.
Note: No phenotypes present.
--score: 14365 valid predictors loaded.
--score: 17 ranges processed.
Results written to tmp_results.*.profile.
PRS is generated!
The PRS result stores in .prs_validation['double_weight']!
--- Done in 0:00:00 ---
The best fit p-value is 0.00000001
The best fit result is stored in
processor.prs_validation['double_weight']['best_fit']
The best fit result is stored in
processor.tuning['double_weight']['coef_squared']
Extracting adjusted sumstats from double_weight method...
Generating PRS...

Warning: 1 line skipped in --q-score-range data file.

PLINK v1.90b6.21 64-bit (19 Oct 2020)          www.cog-genomics.org/plink/1.9/
(C) 2005-2020 Shaun Purcell, Christopher Chang   GNU General Public License v3
Logging to tmp_results.log.
Options in effect:
  --bfile workdir_UKBB_tutorial/preprocessed_test
  --out tmp_results
  --q-score-range tmp_range_list tmp_SNP.pvalue
  --score workdir_UKBB_tutorial/adjusted_sumstats_double_weight 3 4 9 header sum

31807 MB RAM detected; reserving 15903 MB for main workspace.
22331 variants loaded from .bim file.
257 people (0 males, 0 females, 257 ambiguous) loaded from .fam.
Ambiguous sex IDs written to tmp_results.nosex .
Using 1 thread (no multithreaded calculations invoked).
Before main variant filters, 257 founders and 0 nonfounders present.
Calculating allele frequencies... 10111213141516171819202122232425262728293031 32
33343536373839404142434445464748495051525354555657585960616263646566676869707172
73747576777879808182838485868788899091929394959697989 done.
22331 variants and 257 people pass filters and QC.
Note: No phenotypes present.
--score: 14365 valid predictors loaded.
--score: 1 range processed.
Results written to tmp_results.*.profile.
PRS is generated!
The PRS result stores in .prs_test['double_weight']!
--- Done in 0:00:00 ---
The best fit p-value is 0.00000001
The best fit result is stored in processor.prs_test['double_weight']['best_fit']
```

```
The best fit result is stored in
processor.performance['double_weight']['coef_squared']

Warning: 1 line skipped in --q-score-range data file.
```

1.0.6 Running PRS models for VN population (change the sumstats)

[6]:
```
processor_VN = tprs.read_input(prefix_test="tutorials/data/test_demo",
                test_phenotype="tutorials/data/test_demo.phenotype",
                sumstats_path="tutorials/data/VN_train_sumstats.ss",
                prefix_validation="tutorials/data/val_demo",
                validation_phenotype="tutorials/data/validation_demo.phenotype",
                workdir="workdir_VN_tutorial")
```

```
Reading the Test genotype...

Mapping files: 100%|| 3/3
[00:00<00:00, 82.00it/s]

Reading the Validation genotype...

Mapping files: 100%|| 3/3
[00:00<00:00, 95.77it/s]

Phenotype stored in .phenotype
Phenotype stored in .phenotype_val
```

[7]:
```
tprs.Preprocessing(processor_VN, n_components=6)
```

```
PLINK v1.90b6.21 64-bit (19 Oct 2020)          www.cog-genomics.org/plink/1.9/
(C) 2005-2020 Shaun Purcell, Christopher Chang   GNU General Public License v3
Logging to tmp.log.
Options in effect:
--bfile tmp
--indep-pairwise 200 50 0.25
--out tmp

31807 MB RAM detected; reserving 15903 MB for main workspace.
22331 variants loaded from .bim file.
257 people (0 males, 0 females, 257 ambiguous) loaded from .fam.
Ambiguous sex IDs written to tmp.nosex .
Using 1 thread (no multithreaded calculations invoked).
Before main variant filters, 257 founders and 0 nonfounders present.
Calculating allele frequencies... 10111213141516171819202122232425262728293031323334353637383940414243444546474849505152535455565758596061626364656667686970717273747576777879808182838485868788899091929394959697989 done.
22331 variants and 257 people pass filters and QC.
Note: No phenotypes present.
Pruned 9950 variants from chromosome 21, leaving 1559.
Pruned 9120 variants from chromosome 22, leaving 1702.
Pruning complete.  19070 of 22331 variants removed.
Marker lists written to tmp.prune.in and tmp.prune.out .
PLINK v1.90b6.21 64-bit (19 Oct 2020)          www.cog-genomics.org/plink/1.9/
(C) 2005-2020 Shaun Purcell, Christopher Chang   GNU General Public License v3
Logging to tmp.log.
Options in effect:
--bfile tmp
--extract tmp.prune.in
```

```
--out tmp
--pca 6

31807 MB RAM detected; reserving 15903 MB for main workspace.
22331 variants loaded from .bim file.
257 people (0 males, 0 females, 257 ambiguous) loaded from .fam.
Ambiguous sex IDs written to tmp.nosex .
--extract: 3261 variants remaining.
Using up to 11 threads (change this with --threads).
Before main variant filters, 257 founders and 0 nonfounders present.
Calculating allele frequencies... 10111213141516171819202122232425262728293031 32
33343536373839404142434445464748495051525354555657585960616263646566676869707172
73747576777879808182838485868788899091929394959697989 done.
3261 variants and 257 people pass filters and QC.
Note: No phenotypes present.
Relationship matrix calculation complete.
--pca: Results saved to tmp.eigenval and tmp.eigenvec .
PCA result is stored in .phenotype
PLINK v1.90b6.21 64-bit (19 Oct 2020)          www.cog-genomics.org/plink/1.9/
(C) 2005-2020 Shaun Purcell, Christopher Chang   GNU General Public License v3
Logging to tmp.log.
Options in effect:
--bfile tmp
--indep-pairwise 200 50 0.25
--out tmp

31807 MB RAM detected; reserving 15903 MB for main workspace.
22331 variants loaded from .bim file.
258 people (0 males, 0 females, 258 ambiguous) loaded from .fam.
Ambiguous sex IDs written to tmp.nosex .
Using 1 thread (no multithreaded calculations invoked).
Before main variant filters, 258 founders and 0 nonfounders present.
Calculating allele frequencies... 10111213141516171819202122232425262728293031 32
33343536373839404142434445464748495051525354555657585960616263646566676869707172
73747576777879808182838485868788899091929394959697989 done.
22331 variants and 258 people pass filters and QC.
Note: No phenotypes present.
Pruned 9958 variants from chromosome 21, leaving 1551.
Pruned 9129 variants from chromosome 22, leaving 1693.
Pruning complete.  19087 of 22331 variants removed.
Marker lists written to tmp.prune.in and tmp.prune.out .
PLINK v1.90b6.21 64-bit (19 Oct 2020)          www.cog-genomics.org/plink/1.9/
(C) 2005-2020 Shaun Purcell, Christopher Chang   GNU General Public License v3
Logging to tmp.log.
Options in effect:
--bfile tmp
--extract tmp.prune.in
--out tmp
--pca 6

31807 MB RAM detected; reserving 15903 MB for main workspace.
22331 variants loaded from .bim file.
258 people (0 males, 0 females, 258 ambiguous) loaded from .fam.
Ambiguous sex IDs written to tmp.nosex .
```

```
--extract: 3244 variants remaining.
Using up to 11 threads (change this with --threads).
Before main variant filters, 258 founders and 0 nonfounders present.
Calculating allele frequencies... 10111213141516171819202122232425262728293031 32
33343536373839404142434445464748495051525354555657585960616263646566676869707172
73747576777879808182838485868788899091929394959697989 done.
3244 variants and 258 people pass filters and QC.
Note: No phenotypes present.
Relationship matrix calculation complete.
--pca: Results saved to tmp.eigenval and tmp.eigenvec .
PCA result is stored in .phenotype
Created folder workdir_VN_tutorial for working directory
Stored sumstats workdir_VN_tutorial/preprocessed_sumstats in working directory
Stored test genotype workdir_VN_tutorial/preprocessed_test in working directory
Stored validation genotype workdir_VN_tutorial/preprocessed_test in working
directory
Stored test genotype workdir_VN_tutorial/preprocessed_test in working directory
```

[8]:
```
# Run P+T
tprs.methods.clumping(processor_VN)

tprs.scoring.generate_prs(processor_VN,method="clumping")

tprs.metrics.coef_squared_evaluation(processor_VN,
                                     method="clumping",
                                     trait_col="Height",
                                     prs_col="SCORESUM",
                                    )

tprs.scoring.generate_prs(processor_VN,method="clumping",validate=False)

tprs.metrics.coef_squared_evaluation(processor_VN,
                                     method="clumping",
                                     trait_col="Height",
                                     prs_col="SCORESUM",
                                    validate=False)
```

```
Clumping is running...
PLINK v1.90b6.21 64-bit (19 Oct 2020)          www.cog-genomics.org/plink/1.9/
(C) 2005-2020 Shaun Purcell, Christopher Chang   GNU General Public License v3
Logging to tmp_out.log.
Options in effect:
--bfile workdir_VN_tutorial/preprocessed_validation
--clump workdir_VN_tutorial/preprocessed_sumstats
--clump-field P
--clump-kb 250
--clump-p1 1
--clump-r2 0.5
--clump-snp-field SNP
--out tmp_out

31807 MB RAM detected; reserving 15903 MB for main workspace.
22331 variants loaded from .bim file.
258 people (0 males, 0 females, 258 ambiguous) loaded from .fam.
```

```
Ambiguous sex IDs written to tmp_out.nosex .
Using 1 thread (no multithreaded calculations invoked).
Before main variant filters, 258 founders and 0 nonfounders present.
Calculating allele frequencies... 10111213141516171819202122232425262728293031323334353637383940414243444546474849505152535455565758596061626364656667686970717273747576777879808182838485868788899091929394959697989 done.
22331 variants and 258 people pass filters and QC.
Note: No phenotypes present.
--clump: 6281 clumps formed from 21616 top variants.
Results written to tmp_out.clumped .
Done clumping!
The clumping result stores in .adjusted_ss['clumping']!
--- Done in 0:00:01 ---
Extracting adjusted sumstats from clumping method...
Generating PRS...
PLINK v1.90b6.21 64-bit (19 Oct 2020)          www.cog-genomics.org/plink/1.9/
(C) 2005-2020 Shaun Purcell, Christopher Chang   GNU General Public License v3
Logging to tmp_results.log.
Options in effect:
  --bfile workdir_VN_tutorial/preprocessed_validation
  --out tmp_results
  --q-score-range tmp_range_list tmp_SNP.pvalue
  --score workdir_VN_tutorial/adjusted_sumstats_clumping 3 4 9 header sum

31807 MB RAM detected; reserving 15903 MB for main workspace.
22331 variants loaded from .bim file.
258 people (0 males, 0 females, 258 ambiguous) loaded from .fam.
Ambiguous sex IDs written to tmp_results.nosex .
Using 1 thread (no multithreaded calculations invoked).
Before main variant filters, 258 founders and 0 nonfounders present.
Calculating allele frequencies... 10111213141516171819202122232425262728293031323334353637383940414243444546474849505152535455565758596061626364656667686970717273747576777879808182838485868788899091929394959697989 done.
22331 variants and 258 people pass filters and QC.
Note: No phenotypes present.
--score: 6281 valid predictors loaded.
--score: 9 ranges processed (8 empty ranges skipped).
Results written to tmp_results.*.profile.
PRS is generated!
The PRS result stores in .prs_validation['clumping']!
--- Done in 0:00:00 ---
The best fit p-value is 0.001
The best fit result is stored in
processor.prs_validation['clumping']['best_fit']
The best fit result is stored in processor.tuning['clumping']['coef_squared']
Extracting adjusted sumstats from clumping method...
Generating PRS...
PLINK v1.90b6.21 64-bit (19 Oct 2020)          www.cog-genomics.org/plink/1.9/
(C) 2005-2020 Shaun Purcell, Christopher Chang   GNU General Public License v3
Logging to tmp_results.log.
Options in effect:
  --bfile workdir_VN_tutorial/preprocessed_test
  --out tmp_results
  --q-score-range tmp_range_list tmp_SNP.pvalue
```

```
  --score workdir_VN_tutorial/adjusted_sumstats_clumping 3 4 9 header sum

31807 MB RAM detected; reserving 15903 MB for main workspace.
22331 variants loaded from .bim file.
257 people (0 males, 0 females, 257 ambiguous) loaded from .fam.
Ambiguous sex IDs written to tmp_results.nosex .
Using 1 thread (no multithreaded calculations invoked).
Before main variant filters, 257 founders and 0 nonfounders present.
Calculating allele frequencies... 10111213141516171819202122232425262728293031 32
33343536373839404142434445464748495051525354555657585960616263646566676869707172
73747576777879808182838485868788899091929394959697989 done.
22331 variants and 257 people pass filters and QC.
Note: No phenotypes present.
--score: 6281 valid predictors loaded.
--score: 1 range processed.
Results written to tmp_results.*.profile.
PRS is generated!
The PRS result stores in .prs_test['clumping']!
--- Done in 0:00:00 ---
The best fit p-value is 0.001
The best fit result is stored in processor.prs_test['clumping']['best_fit']
The best fit result is stored in
processor.performance['clumping']['coef_squared']

Warning: 1 line skipped in --q-score-range data file.
Warning: 1 line skipped in --q-score-range data file.
```

[9]:
```
# Run double weight
tprs.methods.double_weight(processor_VN)

tprs.scoring.generate_prs(processor_VN,method="double_weight")

tprs.metrics.coef_squared_evaluation(processor_VN,
                                     method="double_weight",
                                     trait_col="Height",
                                     prs_col="SCORESUM",
                                    )

tprs.scoring.generate_prs(processor_VN,method="double_weight",validate=False)

tprs.metrics.coef_squared_evaluation(processor_VN,
                                     method="double_weight",
                                     trait_col="Height",
                                     prs_col="SCORESUM",
                                    validate=False)
```

```
Double weight method is running...
[1] "Adjusted BETA is done!"
Done Double weight!
The double weight result stores in .adjusted_ss['double_weight']!
--- Done in 0:00:01 ---
Extracting adjusted sumstats from double_weight method...
Generating PRS...
PLINK v1.90b6.21 64-bit (19 Oct 2020)          www.cog-genomics.org/plink/1.9/
(C) 2005-2020 Shaun Purcell, Christopher Chang   GNU General Public License v3
```

```
Logging to tmp_results.log.
Options in effect:
  --bfile workdir_VN_tutorial/preprocessed_validation
  --out tmp_results
  --q-score-range tmp_range_list tmp_SNP.pvalue
  --score workdir_VN_tutorial/adjusted_sumstats_double_weight 3 4 9 header sum

31807 MB RAM detected; reserving 15903 MB for main workspace.
22331 variants loaded from .bim file.
258 people (0 males, 0 females, 258 ambiguous) loaded from .fam.
Ambiguous sex IDs written to tmp_results.nosex .
Using 1 thread (no multithreaded calculations invoked).
Before main variant filters, 258 founders and 0 nonfounders present.
Calculating allele frequencies... 10111213141516171819202122232425262728293031 32
33343536373839404142434445464748495051525354555657585960616263646566676869707172
73747576777879808182838485868788899091929394959697989 done.
22331 variants and 258 people pass filters and QC.
Note: No phenotypes present.
--score: 21616 valid predictors loaded.
--score: 9 ranges processed (8 empty ranges skipped).
Results written to tmp_results.*.profile.
PRS is generated!
The PRS result stores in .prs_validation['double_weight']!
--- Done in 0:00:00 ---
The best fit p-value is 0.001
The best fit result is stored in
processor.prs_validation['double_weight']['best_fit']
The best fit result is stored in
processor.tuning['double_weight']['coef_squared']
Extracting adjusted sumstats from double_weight method...
Generating PRS...
PLINK v1.90b6.21 64-bit (19 Oct 2020)          www.cog-genomics.org/plink/1.9/
(C) 2005-2020 Shaun Purcell, Christopher Chang   GNU General Public License v3
Logging to tmp_results.log.
Options in effect:
  --bfile workdir_VN_tutorial/preprocessed_test
  --out tmp_results
  --q-score-range tmp_range_list tmp_SNP.pvalue
  --score workdir_VN_tutorial/adjusted_sumstats_double_weight 3 4 9 header sum

31807 MB RAM detected; reserving 15903 MB for main workspace.
22331 variants loaded from .bim file.
257 people (0 males, 0 females, 257 ambiguous) loaded from .fam.
Ambiguous sex IDs written to tmp_results.nosex .
Using 1 thread (no multithreaded calculations invoked).
Before main variant filters, 257 founders and 0 nonfounders present.
Calculating allele frequencies... 10111213141516171819202122232425262728293031 32
33343536373839404142434445464748495051525354555657585960616263646566676869707172
73747576777879808182838485868788899091929394959697989 done.
22331 variants and 257 people pass filters and QC.
Note: No phenotypes present.
--score: 21616 valid predictors loaded.
--score: 1 range processed.
Results written to tmp_results.*.profile.
```

```
PRS is generated!
The PRS result stores in .prs_test['double_weight']!
--- Done in 0:00:00 ---
The best fit p-value is 0.001
The best fit result is stored in processor.prs_test['double_weight']['best_fit']
The best fit result is stored in
processor.performance['double_weight']['coef_squared']

Warning: 1 line skipped in --q-score-range data file.
Warning: 1 line skipped in --q-score-range data file.
```

1.0.7 Running PRS models for JPN population (change the sumstats)

[10]:
```
processor_JPN = tprs.read_input(prefix_test="tutorials/data/test_demo",
                test_phenotype="tutorials/data/test_demo.phenotype",
                sumstats_path="tutorials/data/JPN_height_sumstat.ss",
                prefix_validation="tutorials/data/val_demo",
                validation_phenotype="tutorials/data/validation_demo.phenotype",
                workdir="workdir_JPN_tutorial")
```

```
Reading the Test genotype...

Mapping files: 100%|| 3/3
[00:00<00:00, 78.33it/s]

Reading the Validation genotype...

Mapping files: 100%|| 3/3
[00:00<00:00, 94.32it/s]

Phenotype stored in .phenotype
Phenotype stored in .phenotype_val
```

[11]:
```
tprs.Preprocessing(processor_JPN, n_components=6)
```

```
PLINK v1.90b6.21 64-bit (19 Oct 2020)          www.cog-genomics.org/plink/1.9/
(C) 2005-2020 Shaun Purcell, Christopher Chang   GNU General Public License v3
Logging to tmp.log.
Options in effect:
--bfile tmp
--indep-pairwise 200 50 0.25
--out tmp

31807 MB RAM detected; reserving 15903 MB for main workspace.
22331 variants loaded from .bim file.
257 people (0 males, 0 females, 257 ambiguous) loaded from .fam.
Ambiguous sex IDs written to tmp.nosex .
Using 1 thread (no multithreaded calculations invoked).
Before main variant filters, 257 founders and 0 nonfounders present.
Calculating allele frequencies... 10111213141516171819202122232425262728293031323334353637383940414243444546474849505152535455565758596061626364656667686970717273747576777879808182838485868788899091929394959697989 done.
22331 variants and 257 people pass filters and QC.
Note: No phenotypes present.
Pruned 9950 variants from chromosome 21, leaving 1559.
Pruned 9120 variants from chromosome 22, leaving 1702.
Pruning complete.  19070 of 22331 variants removed.
Marker lists written to tmp.prune.in and tmp.prune.out .
```

```
PLINK v1.90b6.21 64-bit (19 Oct 2020)          www.cog-genomics.org/plink/1.9/
(C) 2005-2020 Shaun Purcell, Christopher Chang   GNU General Public License v3
Logging to tmp.log.
Options in effect:
--bfile tmp
--extract tmp.prune.in
--out tmp
--pca 6

31807 MB RAM detected; reserving 15903 MB for main workspace.
22331 variants loaded from .bim file.
257 people (0 males, 0 females, 257 ambiguous) loaded from .fam.
Ambiguous sex IDs written to tmp.nosex .
--extract: 3261 variants remaining.
Using up to 11 threads (change this with --threads).
Before main variant filters, 257 founders and 0 nonfounders present.
Calculating allele frequencies... 10111213141516171819202122232425262728293031 32
33343536373839404142434445464748495051525354555657585960616263646566676869707172
73747576777879808182838485868788899091929394959697989 done.
3261 variants and 257 people pass filters and QC.
Note: No phenotypes present.
Relationship matrix calculation complete.
--pca: Results saved to tmp.eigenval and tmp.eigenvec .
PCA result is stored in .phenotype
PLINK v1.90b6.21 64-bit (19 Oct 2020)          www.cog-genomics.org/plink/1.9/
(C) 2005-2020 Shaun Purcell, Christopher Chang   GNU General Public License v3
Logging to tmp.log.
Options in effect:
--bfile tmp
--indep-pairwise 200 50 0.25
--out tmp

31807 MB RAM detected; reserving 15903 MB for main workspace.
22331 variants loaded from .bim file.
258 people (0 males, 0 females, 258 ambiguous) loaded from .fam.
Ambiguous sex IDs written to tmp.nosex .
Using 1 thread (no multithreaded calculations invoked).
Before main variant filters, 258 founders and 0 nonfounders present.
Calculating allele frequencies... 10111213141516171819202122232425262728293031 32
33343536373839404142434445464748495051525354555657585960616263646566676869707172
73747576777879808182838485868788899091929394959697989 done.
22331 variants and 258 people pass filters and QC.
Note: No phenotypes present.
Pruned 9958 variants from chromosome 21, leaving 1551.
Pruned 9129 variants from chromosome 22, leaving 1693.
Pruning complete.  19087 of 22331 variants removed.
Marker lists written to tmp.prune.in and tmp.prune.out .
PLINK v1.90b6.21 64-bit (19 Oct 2020)          www.cog-genomics.org/plink/1.9/
(C) 2005-2020 Shaun Purcell, Christopher Chang   GNU General Public License v3
Logging to tmp.log.
Options in effect:
--bfile tmp
--extract tmp.prune.in
--out tmp
```

```
--pca 6

31807 MB RAM detected; reserving 15903 MB for main workspace.
22331 variants loaded from .bim file.
258 people (0 males, 0 females, 258 ambiguous) loaded from .fam.
Ambiguous sex IDs written to tmp.nosex .
--extract: 3244 variants remaining.
Using up to 11 threads (change this with --threads).
Before main variant filters, 258 founders and 0 nonfounders present.
Calculating allele frequencies... 10111213141516171819202122232425262728293031323
33343536373839404142434445464748495051525354555657585960616263646566676869707172
73747576777879808182838485868788899091929394959697989 done.
3244 variants and 258 people pass filters and QC.
Note: No phenotypes present.
Relationship matrix calculation complete.
--pca: Results saved to tmp.eigenval and tmp.eigenvec .
PCA result is stored in .phenotype
Created folder workdir_JPN_tutorial for working directory
Stored sumstats workdir_JPN_tutorial/preprocessed_sumstats in working directory
Stored test genotype workdir_JPN_tutorial/preprocessed_test in working directory
Stored validation genotype workdir_JPN_tutorial/preprocessed_test in working
directory
Stored test genotype workdir_JPN_tutorial/preprocessed_test in working directory
```

[12]:
```
# Run P+T
tprs.methods.clumping(processor_JPN)

tprs.scoring.generate_prs(processor_JPN,method="clumping")

tprs.metrics.coef_squared_evaluation(processor_JPN,
                                     method="clumping",
                                     trait_col="Height",
                                     prs_col="SCORESUM",
                                    )

tprs.scoring.generate_prs(processor_JPN,method="clumping",validate=False)

tprs.metrics.coef_squared_evaluation(processor_JPN,
                                     method="clumping",
                                     trait_col="Height",
                                     prs_col="SCORESUM",
                                    validate=False)
```

```
Clumping is running...
PLINK v1.90b6.21 64-bit (19 Oct 2020)          www.cog-genomics.org/plink/1.9/
(C) 2005-2020 Shaun Purcell, Christopher Chang   GNU General Public License v3
Logging to tmp_out.log.
Options in effect:
--bfile workdir_JPN_tutorial/preprocessed_validation
--clump workdir_JPN_tutorial/preprocessed_sumstats
--clump-field P
--clump-kb 250
--clump-p1 1
--clump-r2 0.5
```

```
--clump-snp-field SNP
--out tmp_out

31807 MB RAM detected; reserving 15903 MB for main workspace.
22331 variants loaded from .bim file.
258 people (0 males, 0 females, 258 ambiguous) loaded from .fam.
Ambiguous sex IDs written to tmp_out.nosex .
Using 1 thread (no multithreaded calculations invoked).
Before main variant filters, 258 founders and 0 nonfounders present.
Calculating allele frequencies... 10111213141516171819202122232425262728293031323334353637383940414243444546474849505152535455565758596061626364656667686970717273747576777879808182838485868788899091929394959697989 done.
22331 variants and 258 people pass filters and QC.
Note: No phenotypes present.
--clump: 6338 clumps formed from 21934 top variants.
Results written to tmp_out.clumped .
Done clumping!
The clumping result stores in .adjusted_ss['clumping']!
--- Done in 0:00:01 ---
Extracting adjusted sumstats from clumping method...
Generating PRS...
PLINK v1.90b6.21 64-bit (19 Oct 2020)          www.cog-genomics.org/plink/1.9/
(C) 2005-2020 Shaun Purcell, Christopher Chang   GNU General Public License v3
Logging to tmp_results.log.
Options in effect:
  --bfile workdir_JPN_tutorial/preprocessed_validation
  --out tmp_results
  --q-score-range tmp_range_list tmp_SNP.pvalue
  --score workdir_JPN_tutorial/adjusted_sumstats_clumping 3 4 9 header sum

31807 MB RAM detected; reserving 15903 MB for main workspace.
22331 variants loaded from .bim file.
258 people (0 males, 0 females, 258 ambiguous) loaded from .fam.
Ambiguous sex IDs written to tmp_results.nosex .
Using 1 thread (no multithreaded calculations invoked).
Before main variant filters, 258 founders and 0 nonfounders present.
Calculating allele frequencies... 10111213141516171819202122232425262728293031323334353637383940414243444546474849505152535455565758596061626364656667686970717273747576777879808182838485868788899091929394959697989 done.
22331 variants and 258 people pass filters and QC.
Note: No phenotypes present.
--score: 6338 valid predictors loaded.
--score: 17 ranges processed.
Results written to tmp_results.*.profile.
PRS is generated!
The PRS result stores in .prs_validation['clumping']!
--- Done in 0:00:00 ---
The best fit p-value is 0.000000001
The best fit result is stored in
processor.prs_validation['clumping']['best_fit']
The best fit result is stored in processor.tuning['clumping']['coef_squared']
Extracting adjusted sumstats from clumping method...
Generating PRS...
PLINK v1.90b6.21 64-bit (19 Oct 2020)          www.cog-genomics.org/plink/1.9/
```

```
(C) 2005-2020 Shaun Purcell, Christopher Chang   GNU General Public License v3
Logging to tmp_results.log.
Options in effect:
  --bfile workdir_JPN_tutorial/preprocessed_test
  --out tmp_results
  --q-score-range tmp_range_list tmp_SNP.pvalue
  --score workdir_JPN_tutorial/adjusted_sumstats_clumping 3 4 9 header sum

31807 MB RAM detected; reserving 15903 MB for main workspace.
22331 variants loaded from .bim file.
257 people (0 males, 0 females, 257 ambiguous) loaded from .fam.
Ambiguous sex IDs written to tmp_results.nosex .
Using 1 thread (no multithreaded calculations invoked).
Before main variant filters, 257 founders and 0 nonfounders present.
Calculating allele frequencies... 10111213141516171819202122232425262728293031323334353637383940414243444546474849505152535455565758596061626364656667686970717273747576777879808182838485868788899091929394959697989 done.
22331 variants and 257 people pass filters and QC.
Note: No phenotypes present.
--score: 6338 valid predictors loaded.
--score: 1 range processed.
Results written to tmp_results.*.profile.
PRS is generated!
The PRS result stores in .prs_test['clumping']!
--- Done in 0:00:00 ---
The best fit p-value is 0.000000001
The best fit result is stored in processor.prs_test['clumping']['best_fit']
The best fit result is stored in
processor.performance['clumping']['coef_squared']

Warning: 1 line skipped in --q-score-range data file.
Warning: 1 line skipped in --q-score-range data file.
```

[13]:
```
# Run double weight
tprs.methods.double_weight(processor_JPN)

tprs.scoring.generate_prs(processor_JPN,method="double_weight")

tprs.metrics.coef_squared_evaluation(processor_JPN,
                                     method="double_weight",
                                     trait_col="Height",
                                     prs_col="SCORESUM",
                                    )

tprs.scoring.generate_prs(processor_JPN,method="double_weight",validate=False)

tprs.metrics.coef_squared_evaluation(processor_JPN,
                                     method="double_weight",
                                     trait_col="Height",
                                     prs_col="SCORESUM",
                                    validate=False)
```

```
Double weight method is running...
[1] "Adjusted BETA is done!"
Done Double weight!
```

```
The double weight result stores in .adjusted_ss['double_weight']!
--- Done in 0:00:01 ---
Extracting adjusted sumstats from double_weight method...
Generating PRS...
PLINK v1.90b6.21 64-bit (19 Oct 2020)          www.cog-genomics.org/plink/1.9/
(C) 2005-2020 Shaun Purcell, Christopher Chang   GNU General Public License v3
Logging to tmp_results.log.
Options in effect:
  --bfile workdir_JPN_tutorial/preprocessed_validation
  --out tmp_results
  --q-score-range tmp_range_list tmp_SNP.pvalue
  --score workdir_JPN_tutorial/adjusted_sumstats_double_weight 3 4 9 header sum

31807 MB RAM detected; reserving 15903 MB for main workspace.
22331 variants loaded from .bim file.
258 people (0 males, 0 females, 258 ambiguous) loaded from .fam.
Ambiguous sex IDs written to tmp_results.nosex .
Using 1 thread (no multithreaded calculations invoked).
Before main variant filters, 258 founders and 0 nonfounders present.
Calculating allele frequencies... 10111213141516171819202122232425262728293031323
33343536373839404142434445464748495051525354555657585960616263646566676869707172
73747576777879808182838485868788899091929394959697989 done.
22331 variants and 258 people pass filters and QC.
Note: No phenotypes present.
--score: 21934 valid predictors loaded.
--score: 17 ranges processed.
Results written to tmp_results.*.profile.
PRS is generated!
The PRS result stores in .prs_validation['double_weight']!
--- Done in 0:00:00 ---
The best fit p-value is 0.000000001
The best fit result is stored in
processor.prs_validation['double_weight']['best_fit']
The best fit result is stored in
processor.tuning['double_weight']['coef_squared']
Extracting adjusted sumstats from double_weight method...
Generating PRS...

Warning: 1 line skipped in --q-score-range data file.
Warning: 1 line skipped in --q-score-range data file.

PLINK v1.90b6.21 64-bit (19 Oct 2020)          www.cog-genomics.org/plink/1.9/
(C) 2005-2020 Shaun Purcell, Christopher Chang   GNU General Public License v3
Logging to tmp_results.log.
Options in effect:
  --bfile workdir_JPN_tutorial/preprocessed_test
  --out tmp_results
  --q-score-range tmp_range_list tmp_SNP.pvalue
  --score workdir_JPN_tutorial/adjusted_sumstats_double_weight 3 4 9 header sum

31807 MB RAM detected; reserving 15903 MB for main workspace.
22331 variants loaded from .bim file.
257 people (0 males, 0 females, 257 ambiguous) loaded from .fam.
Ambiguous sex IDs written to tmp_results.nosex .
Using 1 thread (no multithreaded calculations invoked).
```

```
Before main variant filters, 257 founders and 0 nonfounders present.
Calculating allele frequencies... 10111213141516171819202122232425262728293031323334353637383940414243444546474849505152535455565758596061626364656667686970717273747576777879808182838485868788899091929394959697989 done.
22331 variants and 257 people pass filters and QC.
Note: No phenotypes present.
--score: 21934 valid predictors loaded.
--score: 1 range processed.
Results written to tmp_results.*.profile.
PRS is generated!
The PRS result stores in .prs_test['double_weight']!
--- Done in 0:00:00 ---
The best fit p-value is 0.000000001
The best fit result is stored in processor.prs_test['double_weight']['best_fit']
The best fit result is stored in
processor.performance['double_weight']['coef_squared']
```

1.0.8 Combine populations with multiple populations strategy

In this strategy, we used combination of all methods for each combination. It will be easy to extend the number of population and methods.

The suffix number is the order of population in the list.

[14]:
```
# Set a list of all DataProcessor objects
processors = [processor_UKBB,processor_VN,processor_JPN]
```

[15]:
```
# Set a list of methods user wants to combine
methods = ["clumping","double_weight"]
```

[16]:
```
# Perform the combination with weighting method as linear regression
tprs.Combine_multipop_methods(processors, methods=methods,
                                    trait_col="Height",
                                    prs_col="SCORESUM",
                                    use_col="BETA",
                                    metric="coef_squared")
```

```
Estimating mixing weights...
[ 0.13633192  0.27084535 -0.41189508]
The clumping0+clumping1+clumping2 result stores in
.prs_test['clumping0+clumping1+clumping2']!
--- Done in 0:00:00 ---
The best fit p-value is combine
The best fit result is stored in
processor.prs_test['clumping0+clumping1+clumping2']['best_fit']
The best fit result is stored in
processor.performance['clumping0+clumping1+clumping2']['coef_squared']
Estimating mixing weights...
[ 0.2648689   0.29401207 -0.60476407]
The clumping0+clumping1+double_weight2 result stores in
.prs_test['clumping0+clumping1+double_weight2']!
--- Done in 0:00:00 ---
The best fit p-value is combine
The best fit result is stored in
processor.prs_test['clumping0+clumping1+double_weight2']['best_fit']
The best fit result is stored in
```

```
processor.performance['clumping0+clumping1+double_weight2']['coef_squared']
Estimating mixing weights...
[ 0.1421087   0.42390399 -0.40535884]
The clumping0+double_weight1+clumping2 result stores in
.prs_test['clumping0+double_weight1+clumping2']!
--- Done in 0:00:00 ---
The best fit p-value is combine
The best fit result is stored in
processor.prs_test['clumping0+double_weight1+clumping2']['best_fit']
The best fit result is stored in
processor.performance['clumping0+double_weight1+clumping2']['coef_squared']
Estimating mixing weights...
[ 0.2825132   0.45814572 -0.62027642]
The clumping0+double_weight1+double_weight2 result stores in
.prs_test['clumping0+double_weight1+double_weight2']!
--- Done in 0:00:00 ---
The best fit p-value is combine
The best fit result is stored in
processor.prs_test['clumping0+double_weight1+double_weight2']['best_fit']
The best fit result is stored in
processor.performance['clumping0+double_weight1+double_weight2']['coef_squared']
Estimating mixing weights...
[ 0.23172272  0.25064052 -0.48885158]
The double_weight0+clumping1+clumping2 result stores in
.prs_test['double_weight0+clumping1+clumping2']!
--- Done in 0:00:00 ---
The best fit p-value is combine
The best fit result is stored in
processor.prs_test['double_weight0+clumping1+clumping2']['best_fit']
The best fit result is stored in
processor.performance['double_weight0+clumping1+clumping2']['coef_squared']
Estimating mixing weights...
[ 0.51919066  0.25821754 -0.83761181]
The double_weight0+clumping1+double_weight2 result stores in
.prs_test['double_weight0+clumping1+double_weight2']!
--- Done in 0:00:00 ---
The best fit p-value is combine
The best fit result is stored in
processor.prs_test['double_weight0+clumping1+double_weight2']['best_fit']
The best fit result is stored in
processor.performance['double_weight0+clumping1+double_weight2']['coef_squared']
Estimating mixing weights...
[ 0.21605585  0.4070695  -0.47167282]
The double_weight0+double_weight1+clumping2 result stores in
.prs_test['double_weight0+double_weight1+clumping2']!
--- Done in 0:00:00 ---
The best fit p-value is combine
The best fit result is stored in
processor.prs_test['double_weight0+double_weight1+clumping2']['best_fit']
The best fit result is stored in
processor.performance['double_weight0+double_weight1+clumping2']['coef_squared']
Estimating mixing weights...
[ 0.51505543  0.43093838 -0.84290789]
The double_weight0+double_weight1+double_weight2 result stores in
```

```
.prs_test['double_weight0+double_weight1+double_weight2']!
--- Done in 0:00:00 ---
The best fit p-value is combine
The best fit result is stored in
processor.prs_test['double_weight0+double_weight1+double_weight2']['best_fit']
The best fit result is stored in processor.performance['double_weight0+double_we
ight1+double_weight2']['coef_squared']
```

1.0.9 Visualization

User can use `bar_plot` or `box_plot` to visualize the data

[17]:
```
tprs.visualization.
↪visualize_performance(processor_UKBB,metric="coef_squared",plot_type="bar_plot",cmap="tab20")
```

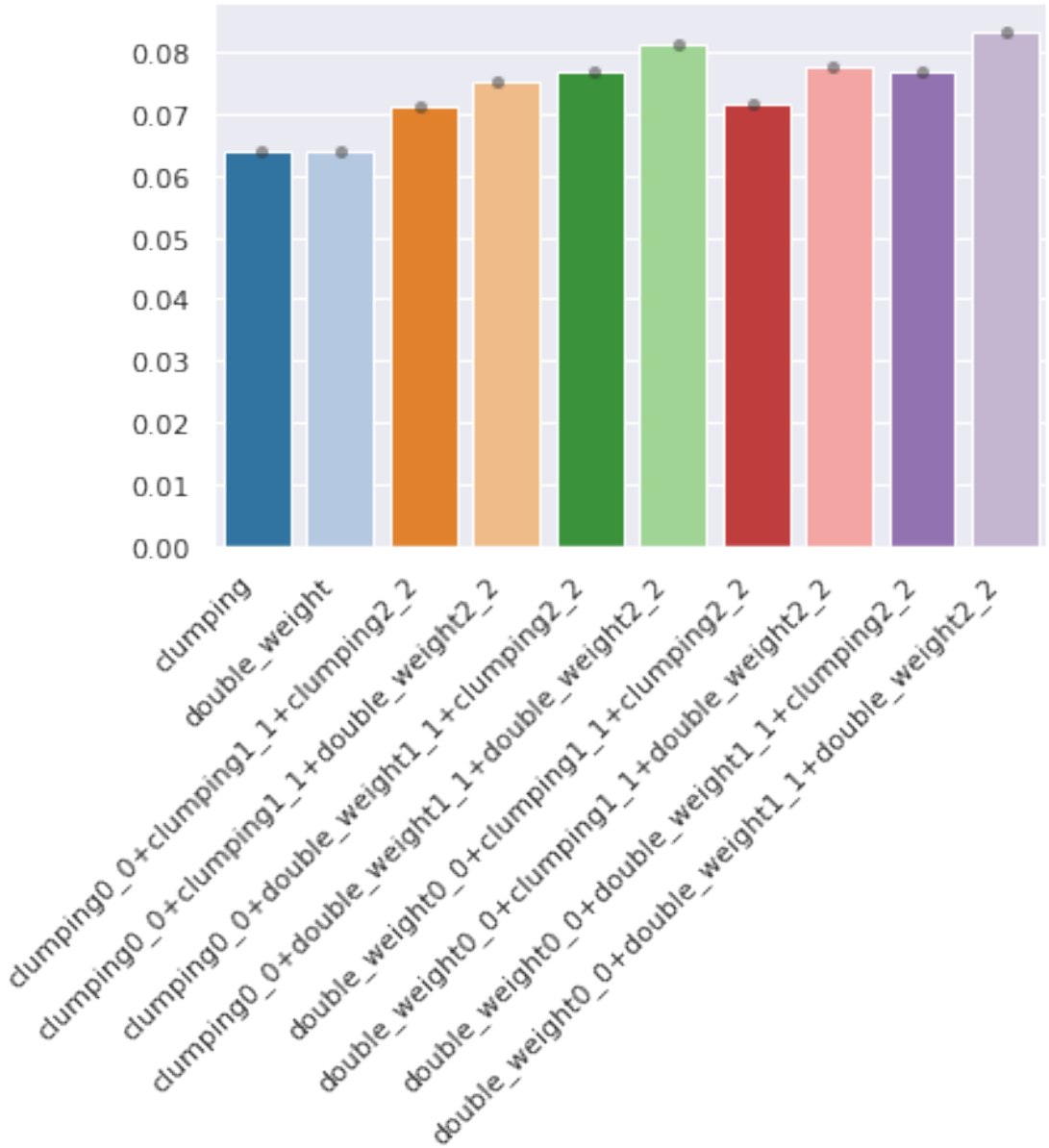

1.0.10 Snippet codes for other methods

PRSCSx Follow the PRSCSx github page https://github.com/getian107/PRScsx to download LD reference and put the path folder to `ldref_dir`

[ ]:
```
tprs.methods.multipop.
  ↪prscsx(processors,["EUR","EAS","EAS"],use_col="BETA",ldref_dir="LD_REF_PATH",N=["300000","800","165056"])

# Result of PRSCSx store in the first DataProcessor in the `processors` list
tprs.scoring.generate_prs(processor_UKBB,method="PRSCSx")

tprs.metrics.coef_squared_evaluation(processor_UKBB,
                                     method="PRSCSx",
                                     trait_col="Height",
                                     prs_col="SCORESUM",
                                    )

tprs.scoring.generate_prs(processor_UKBB,method="PRSCSx",validate=False)

tprs.metrics.coef_squared_evaluation(processor_UKBB,
                                     method="PRSCSx",
                                     trait_col="Height",
                                     prs_col="SCORESUM",
                                    validate=False)
```

PolyFun

[ ]:
```
tprs.methods.polyfun(processor_UKBB, N=300000)

tprs.scoring.generate_prs(processor_UKBB,method="polyfun")

tprs.metrics.coef_squared_evaluation(processor_UKBB,
                                     method="polyfun",
                                     trait_col="Height",
                                     prs_col="SCORESUM",
                                    )

tprs.scoring.generate_prs(processor_UKBB,method="polyfun",validate=False)

tprs.metrics.coef_squared_evaluation(processor_UKBB,
                                     method="polyfun",
                                     trait_col="Height",
                                     prs_col="SCORESUM",
                                    validate=False)
```

SBayesR Access https://cnsgenomics.com/software/gctb/#LDmatrices to download LD reference and put the path folder to `ldm`

[ ]:
```
tprs.methods.SBayesR(processor_UKBB, ldm="LD_REF_PATH/ukb10k.mldm" )

tprs.scoring.generate_prs(processor_UKBB,method="SBayesR")

tprs.metrics.coef_squared_evaluation(processor_UKBB,
                                     method="SBayesR",
                                     trait_col="Height",
                                     prs_col="SCORESUM",
                                    )
```

```
tprs.scoring.generate_prs(processor_UKBB,method="SBayesR",validate=False)

tprs.metrics.coef_squared_evaluation(processor_UKBB,
                                     method="SBayesR",
                                     trait_col="Height",
                                     prs_col="SCORESUM",
                                    validate=False)
```

SBayesS Access https://cnsgenomics.com/software/gctb/#LDmatrices to download LD reference and put the path folder to `ldm`

[ ]:
```
tprs.methods.SBayesS(processor_UKBB, ldm="LD_REF_PATH/ukb10k.mldm")

tprs.scoring.generate_prs(processor_UKBB,method="SBayesS")

tprs.metrics.coef_squared_evaluation(processor_UKBB,
                                     method="SBayesS",
                                     trait_col="Height",
                                     prs_col="SCORESUM",
                                    )

tprs.scoring.generate_prs(processor_UKBB,method="SBayesS",validate=False)

tprs.metrics.coef_squared_evaluation(processor_UKBB,
                                     method="SBayesS",
                                     trait_col="Height",
                                     prs_col="SCORESUM",
                                    validate=False)
```

Ldpred Follow the github page to prepare the reference for LD: https://github.com/bvilhjal/ldpred

[ ]:
```
tprs.methods.ldpred(processor_UKBB,
                    reference="PATH_TO_REF_GENOTYPE",
                    N=754,
                    h2=0.5,
                    fraction_causal=0.02,
                    ldf="REF")

tprs.scoring.generate_prs(processor_UKBB,method="ldpred")

tprs.metrics.coef_squared_evaluation(processor_UKBB,
                                     method="ldpred",
                                     trait_col="Height",
                                     prs_col="SCORESUM",
                                    )

tprs.scoring.generate_prs(processor_UKBB,method="ldpred",validate=False)

tprs.metrics.coef_squared_evaluation(processor_UKBB,
                                     method="ldpred",
                                     trait_col="Height",
                                     prs_col="SCORESUM",
```

```
                                    validate=False)
```

Ldpred2 Follow the homepage to prepare LD reference and the map file: https://privefl.github.io/bigsnpr/articles/LDpred2.html

[ ]:
```
tprs.methods.ldpred2(processor_UKBB,
                     ldref_path = "LD_REF_PATH",
                     map_file = "PATH_TO_MAP/map.rds" )

tprs.scoring.generate_prs(processor_UKBB,method="ldpred2")

tprs.metrics.coef_squared_evaluation(processor_UKBB,
                                     method="ldpred2",
                                     trait_col="Height",
                                     prs_col="SCORESUM",
                                    )

tprs.scoring.generate_prs(processor_UKBB,method="ldpred2",validate=False)

tprs.metrics.coef_squared_evaluation(processor_UKBB,
                                     method="ldpred2",
                                     trait_col="Height",
                                     prs_col="SCORESUM",
                                    validate=False)
```
